# Extreme dry-hot in North America and Europe: the amplified role of warming-enhanced land-air coupling

**DOI:** 10.1093/nsr/nwaf435

**Published:** 2025-10-13

**Authors:** Liang Qiao, Zhiyan Zuo, Renhe Zhang, Wei Mei, Deliang Chen, Meiyu Chang, Kaiwen Zhang

**Affiliations:** Key Laboratory for Semi-Arid Climate Change of the Ministry of Education, College of Atmospheric Sciences, Lanzhou University, Lanzhou 730000, China; Key Laboratory of Polar Atmosphere-ocean-ice System for Weather and Climate of Ministry of Education/Shanghai Key Laboratory of Ocean-Land-Atmosphere Boundary Dynamics and Climate Change, Department of Atmospheric and Oceanic Sciences/Institute of Atmospheric Sciences, Fudan University, Shanghai 200438, China; Key Laboratory of Polar Atmosphere-ocean-ice System for Weather and Climate of Ministry of Education/Shanghai Key Laboratory of Ocean-Land-Atmosphere Boundary Dynamics and Climate Change, Department of Atmospheric and Oceanic Sciences/Institute of Atmospheric Sciences, Fudan University, Shanghai 200438, China; Collaborative Innovation Center on Forecast and Evaluation of Meteorological Disasters, Nanjing University of Information Science and Technology, Nanjing 210044, China; Key Laboratory of Polar Atmosphere-ocean-ice System for Weather and Climate of Ministry of Education/Shanghai Key Laboratory of Ocean-Land-Atmosphere Boundary Dynamics and Climate Change, Department of Atmospheric and Oceanic Sciences/Institute of Atmospheric Sciences, Fudan University, Shanghai 200438, China; Key Laboratory of Polar Atmosphere-ocean-ice System for Weather and Climate of Ministry of Education/Shanghai Key Laboratory of Ocean-Land-Atmosphere Boundary Dynamics and Climate Change, Department of Atmospheric and Oceanic Sciences/Institute of Atmospheric Sciences, Fudan University, Shanghai 200438, China; Department of Earth System Sciences, Tsinghua University, Beijing 100084, China; Department of Earth Sciences, University of Gothenburg, Gothenburg 40530, Sweden; Key Laboratory of Polar Atmosphere-ocean-ice System for Weather and Climate of Ministry of Education/Shanghai Key Laboratory of Ocean-Land-Atmosphere Boundary Dynamics and Climate Change, Department of Atmospheric and Oceanic Sciences/Institute of Atmospheric Sciences, Fudan University, Shanghai 200438, China; Key Laboratory of Polar Atmosphere-ocean-ice System for Weather and Climate of Ministry of Education/Shanghai Key Laboratory of Ocean-Land-Atmosphere Boundary Dynamics and Climate Change, Department of Atmospheric and Oceanic Sciences/Institute of Atmospheric Sciences, Fudan University, Shanghai 200438, China

**Keywords:** land-air coupling, soil moisture, extreme dry-hot, North America and Europe

## Abstract

Greenhouse gases (GHGs) drive global land warming with varying regional impacts, but the role of land-atmosphere interactions in amplifying future warming hotspots remains underexplored. Our study shows that, under uncontrolled GHG emissions, North America and Europe are projected to experience the highest warming by the late 21st century (3.7° ± 0.7°C and 3.8° ± 0.5°C, respectively), exceeding the global average of 2.7° ± 0.4°C in other regions. Approximately one-quarter of this warming in North America and Europe is linked to land-air coupling and associated hot-dry feedback mechanisms, where warming accelerates soil drying, further intensifying surface heating. This feedback could transform nearly 30% of land in these regions into arid or extremely arid zones, significantly impacting ecosystems and agriculture. These results underscore the vulnerability of North America and Europe to amplified climate risks driven by GHG emissions and strengthened land-atmosphere feedbacks.

## Teaser

Warming-enhanced land-air feedbacks could turn North America and Europe into global dry-hot hotspots under uncontrolled greenhouse gases emissions.

## INTRODUCTION

Since the beginning of the Industrial Revolution, anthropogenic emissions of greenhouse gases (GHGs) have resulted in a marked warming of Earth’s climate [1–6]. The Intergovernmental Panel on Climate Change (IPCC) Sixth Assessment Report investigated potential future climate change based on the shared socioeconomic pathways (SSPs) framework, and provided a wide range of plausible societal and climatic futures spanning from a potential below 1.5°C best-estimate warming to over 4°C for 2100 (i.e. SSP1-1.9, SSP1-2.6, SSP2-4.5, SSP3-7.0, and SSP5-8.5) [7]. Among these SSP scenarios, SSP1-2.6 represents a sustainability pathway, by which the global mean surface air temperature (SAT) increases to 2°C above the pre-industrial level [8,9]. For this reason, the SSP1-2.6 scenario can serve as a baseline, for assessing changes in warming, drying, and associated climate risks under other scenarios [10–12].

Although GHGs are uniformly distributed around the globe, their warming effects exhibit considerable regional differences, which are closely related to local feedback [1–4]. For instance, GHG-driven warming over land can trigger a feedback between hotter air and drier soil, further amplifying GHG-induced warming. Existing observations and model simulations both indicate that in land‒air coupling (LAC) hotspots—such as central North America, the Mediterranean coast, and Northeast Asia—warming has been more rapid compared to other regions at the same latitude under modern global warming [3,13–22]. In these regions, LAC enhances incident solar radiation and shifts more land surface heat flux toward sensible heat, which is a key driver of accelerated warming. This LAC-induced warming intensifies the frequency and severity of weather and climate extremes, including heatwaves, droughts, and wildfires [23–32]. Under a high-emission scenario (e.g. SSP5-8.5), this ‘hotter air–drier soil’ feedback could be a crucial driver of additional climate risks over some populous regions in the latter part of this century.

In this study, we show that if GHG emissions were unregulated, North America and Europe in summer would suffer the severest warming and drying in comparison with other regions, using 24 global climate models (Table S1) from the scenario model intercomparison project (ScenarioMIP) [33] within phase 6 of the coupled model intercomparison project (CMIP6). We further show that the intensified warming and drying in the two regions are tied to LAC, by analyzing the land feedback model intercomparison project with prescribed land conditions (LFMIP-pdLC) experiment from the land surface, snow, and soil moisture model intercomparison project (LS3MIP) [34] in CMIP6.

## RESULTS

### Severe warming and drying in hotspots

**Figure 1.**
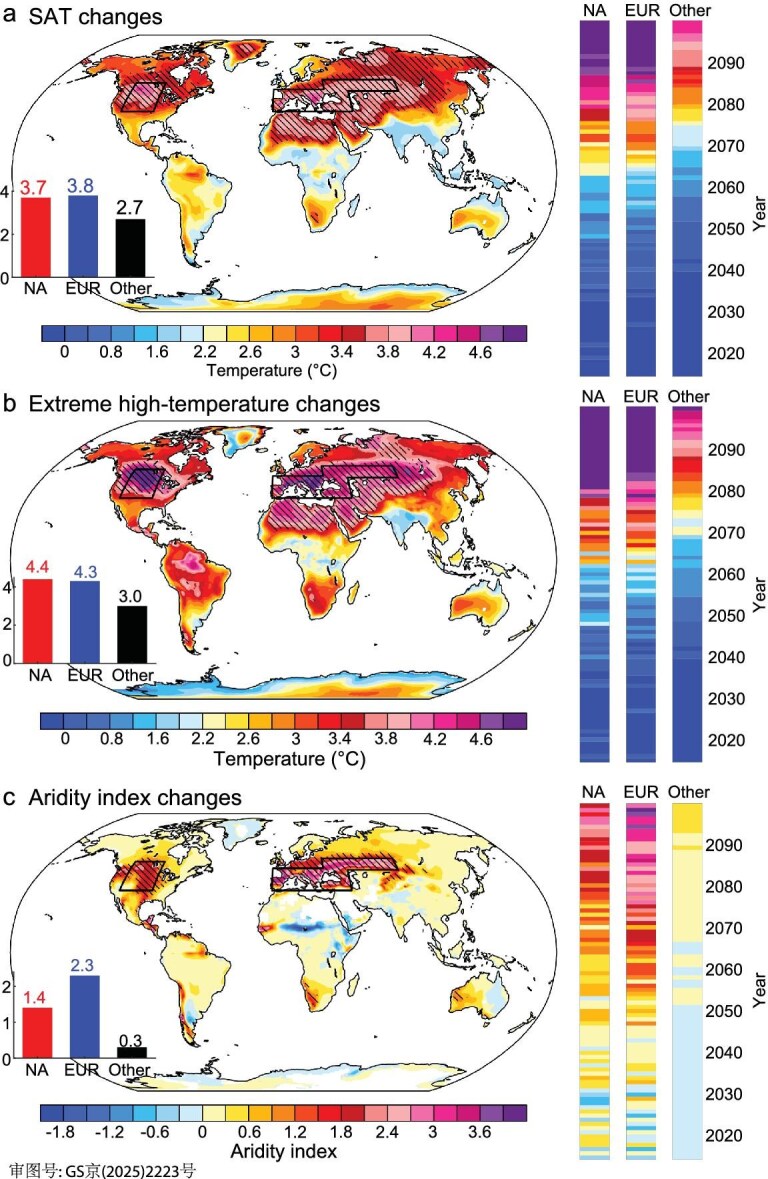
Effect of increased GHG emissions on surface air temperature (SAT), extreme high-temperature, and aridity index. (a) Spatial distribution of the summer SAT (°C) difference between high- and low-emission scenarios (SSP5-8.5 and SSP1-2.6, respectively) for 2060‒2099. Vertical colored strips represent the temporal evolution of summer SAT (°C) difference between high- and low-emission scenarios over central North America (NA: 36°‒53°N, 92°‒115°W), mid-latitudes of western and central Eurasia (EUR: most of Europe and western Siberia, covering [36°‒49°N, 8°W‒30°E], [36°‒55°N, 30°‒48°E], and [48°‒55°N, 48°‒86°E]), and other regions of the world (Other) for 2060‒2099. (b, c) Same as (a), but for extreme high-temperature and aridity index, respectively. Red, blue, and black bars show the latitude-weighted regional averages of SAT, extreme high-temperature, and aridity index over NA, EUR, and Other for 2060‒2099. Black lines delineate the regions of NA and EUR. Areas are marked with stippling where the GHG-driven changes (SSP5-8.5 minus SSP1-2.6) are deemed robust: specifically, where at least 80% of the models project a local change greater than the global land average.

Global land SAT is rising with increased GHG emissions under various scenarios. In comparison with SSP1-2.6, the rate of GHG-driven warming in summer under SSP5-8.5 tends to accelerate after the 2060s (vertical colored strips in Fig. 1a and Fig. S1). The greatest difference in the warming rate occurs in central North America (NA: 36°‒53°N, 92°‒115°W) and mid-latitudes of western and central Eurasia (EUR: most of Europe and western Siberia, covering [36°‒49°N, 8°W‒30°E], [36°‒55°N, 30°‒48°E], and [48°‒55°N, 48°‒86°E]). Specifically, the SAT warming difference between SSP5-8.5 and SSP1-2.6 is projected to be 3.7° ± 0.7°C and 3.8° ± 0.5°C (multi-model mean ± 1σ across models) in NA and EUR during 2060–2099, respectively, compared with 2.7° ± 0.4°C in other regions (shading in Fig. 1a and Fig. S1). As expected, the fully uncontrolled GHG emissions exacerbate the intensity of extreme high-temperatures: the 90 percentile value in NA increases from 36.2° ± 3.4°C in SSP1-2.6 to 40.6° ± 3.1°C in SSP5-8.5, and in EUR increases from 36.1° ± 3.4°C in SSP1-2.6 to 40.4° ± 3.0°C in SSP5-8.5; in other regions the increase is moderate (from 28.4° ± 2.4°C in SSP1-2.6 to 31.4° ± 2.4°C in SSP5-8.5; Fig. 1b).

Meanwhile, fully uncontrolled GHG emissions are also projected to cause more intensive aridity in a non-linear manner over NA and EUR in the late 21st century, while the projected changes in aridity are modest in other regions (Fig. 1c). The aridity index represents the compound effect of precipitation and potential evapotranspiration, which can more exactly reflect the dryness variations of climate regimes in comparison with individual precipitation events [35,36]. An aridity index has been widely used to classify land area into five different types of climate regime: humid, semi-humid, semi-arid, arid, and extremely arid (see Methods) [37,38]. The aridity index differences between SSP5-8.5 and SSP1-2.6 are 1.4 ± 0.7 and 2.3 ± 0.6 over NA and EUR, respectively, but only 0.3 ± 0.1 in other regions. These quantifications of the aridity index suggest that arid regions would additionally increase by 45.9% ± 11.9% over NA and 13.4% ± 6.7% over EUR compared to the sustainability scenario SSP1-2.6 (Fig. S2). Actually, precipitation and soil moisture are also significantly reduced in SSP5-8.5 compared to SSP1-2.6 over NA and EUR (Fig. S3). All individual models consistently exhibit patterns that align with the multi-model mean results for SAT, extreme high-temperature, and aridity index (Figs S4–S9). This robust inter-model agreement strongly supports the conclusion that emission-driven intensification of dry-hot conditions will be particularly pronounced in NA and EUR.

### ‘Hotter air–drier soil’ feedback

**Figure 2.**
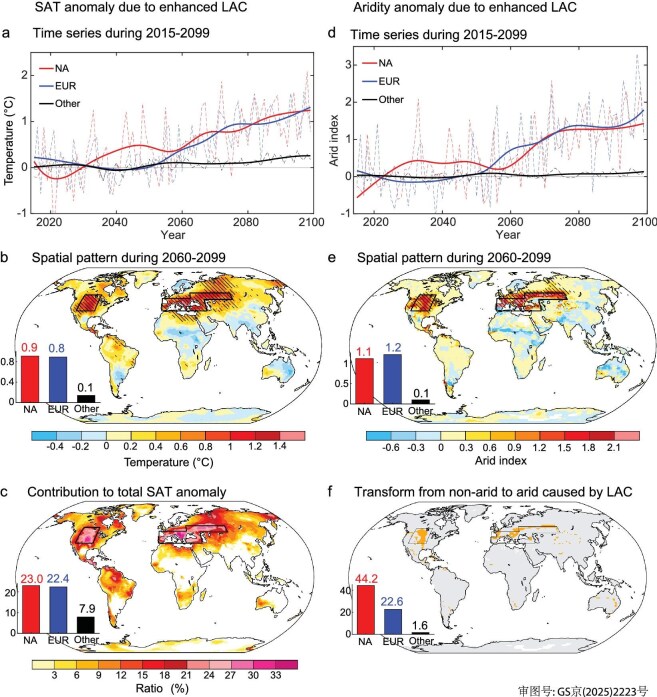
Contribution of land-air coupling (LAC) to warming and drying. (a) Temporal evolution of the difference in the LAC-caused summer SAT (°C) anomaly between SSP585 and SSP126 experiments over NA, EUR, and other regions (Other) during 2060‒2099. The difference is calculated as (SSP585−pdLC585)−(SSP126−pdLC126), where the SSP585 and SSP126 experiments represent fully coupled high- and low-emission scenarios, respectively, and pdLC585 and pdLC126 experiments represent uncoupled high- and low-emission scenarios, respectively. (b) Spatial distribution of the difference during 2060‒2099. (c) Ratio (%) of the difference to the total warming during 2060‒2099: (SSP585−pdLC585)−(SSP126−pdLC126))/(SSP585−SSP126) × 100%. (d, e) Same as (a, b), but for the aridity index. (f) Regions experiencing a shift within 2060‒2099 from non-arid climate condition to arid condition (orange shading in the map), obtained by a comparison between the uncoupled and fully coupled experiments (pdLC585 and SSP585) under high-emission scenario. In (b, c, e, f), black lines delineate the regions of NA and EUR, and the red, blue, and black bars show the latitude-weighted regional averages over NA, EUR, and Other. In (a, d), the fine dashed lines represent the original unsmoothed data, and the thick solid lines show the data after processing with the Ensemble Empirical Mode Decomposition (EEMD) method to remove interannual variability. In (b, e), areas are marked with stippling where the changes in SAT/aridity index exceed the global land average in at least 80% of the models (5+/6).

LAC has been suggested to play a key role in the occurrence of the record-breaking historical heatwaves in NA and EUR [18,19,39–41]. To check and quantify the contribution of LAC to the projected intensification of warming and drying over NA and EUR, we examine the future changes in temperature and aridity in the set of experiments *without* LAC (referred to as pdLC126 and pdLC585; see Methods) and compare them with those in the experiments *with* LAC shown above (referred to as SSP126 and SSP585; Fig. 1). Figure 2a, d displays the temporal evolution of the difference in SAT changes and aridity changes between the two sets of experiments [i.e. (SSP585−pdLC585)−(SSP126−pdLC126)], respectively. The LAC-mediated amplification of GHG-driven warming and drying becomes particularly dramatic over NA and EUR after the 2060s, when the divergence in GHG concentrations between the SSP1-2.6 and SSP5-8.5 scenarios becomes substantial [7].

If GHG emissions are fully uncontrolled, LAC will cause additional warming over most portions of global land (Fig. 2b). This warming is most pronounced over NA and EUR (0.9° ± 0.4°C and 0.8° ± 0.4°C, respectively); in comparison, it is rather modest in other regions (0.1° ± 0.1°C). The LAC-driven warming accounts for around a quarter (23.0% ± 9.8% and 22.4% ± 10.5% for NA and EUR, respectively) of the total temperature differences between experiments SSP585 and SSP126 in the two hotspots, while the contribution is much smaller in other regions (7.9% ± 4.5%; Fig. 2c).

The effect of LAC on making NA and EUR the two hotspots is more significant in terms of aridity (Fig. 2e). Specifically, the LAC-caused increase in the aridity index is 1.1 ± 0.6 and 1.2 ± 0.7 over NA and EUR, respectively; in the remaining regions, the increase is negligible (0.1 ± 0.1). In other words, LAC will increase the percentage of arid and extremely arid regions by 44.2% ± 18.7% (from 16.3% ± 9.9% to 60.5% ± 24.0%) over NA and by 22.6% ± 12.6% (from 70.6% ± 7.6% to 93.2% ± 5.8%) over EUR (29.4% ± 10.9% when the two regions are combined versus 1.6% ± 2.1% in other regions; Fig. 2f, and Fig. S10).

The above quantifications highlight the crucial role of LAC in the drastically unusual warming and drying scenarios over NA and EUR if GHG emissions are fully uncontrolled. Indeed, without the LAC effect, NA and EUR will no longer be the hotspots of warming and drying, with the SAT and aridity increase being substantially suppressed (Fig. S11). Critically, our single-model analyses provide independent reconfirmation that GHG-enhanced LAC preferentially amplifies warming and drying in NA and EUR, with all individual models reproducing this critical pattern (Figs S12–S15).

**Figure 3.**
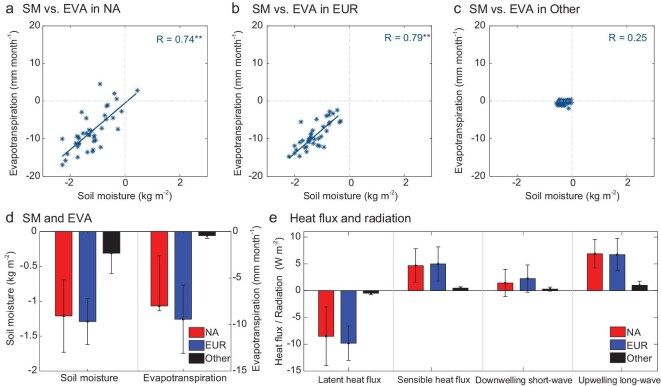
Effects of enhanced LAC on soil moisture, evapotranspiration, and heat fluxes. (a–c) Scatter plot of soil moisture anomaly (SM; kg m^−2^) due to enhanced LAC versus evapotranspiration anomaly (EVA; mm month^−1^) due to enhanced LAC over NA (a), EUR (b), and Other (c) for 2060‒2099. The correlation coefficient between SM and EVA is shown in the top right, with ‘**’ indicating that the correlation coefficient is statistically significant at a 0.01 level. (d) SM (left) and EVA (right) anomalies due to enhanced LAC averaged during 2060‒2099 over NA (red), EUR (blue), and Other (black). (e) Same as (d), but for surface sensible flux, latent heat flux, downwelling short-wave radiation, and upwelling long-wave radiation (W m^−2^), respectively. In (d, e), error bars indicate the spread across models.

Next, we attempt to identify the pathways through which LAC intensifies warming and drying with increasing GHG emissions described above. To achieve this, we examined changes in soil moisture and evapotranspiration, and found that the strengthened LAC due to increased GHG emissions reduces soil moisture by ‒1.2 ± 0.5 and ‒1.3 ± 0.3 kg m^−2^ over NA and EUR, respectively. The reduced soil moisture in turn suppresses evapotranspiration in the two regions by ‒8.0 ± 5.4 and ‒9.4 ± 3.7 mm month^−1^, respectively (Fig. 3a, b, d). In contrast, in other regions, on average, soil moisture and evapotranspiration only decrease by ‒0.3 ± 0.3 kg m^−2^ and ‒0.4 ± 0.3 mm month^−1^, respectively (Fig. 3c, d). The relationship between the changes in soil moisture and changes in evapotranspiration is particularly strong over NA and EUR (the linear correlation coefficient *r* = 0.74 and 0.79, respectively; *p* < 0.01 for both coefficients), in comparison with other regions (*r* = 0.25; *p* > 0.12). Similar results apply to deeper soil layers (for example,the root zone layer at a 100-cm depth; Fig. S16).

In NA and EUR, the marked soil drying trend and evapotranspiration deficit would increase short-wave radiation (1.4 ± 2.5 W m^−2^ over NA and 2.3 ± 2.5 W m^−2^ over EUR) received by the land surface, via reduced cloud cover, and decreased surface latent heat flux (‒8.5 ± 5.5 W m^−2^ over NA and ‒9.8 ± 3.2 W m^−2^ over EUR). These changes would raise ground skin temperature, which in turn enhances upwelling long-wave radiation (6.9 ± 2.7 W m^−2^ over NA and 6.7 ± 3.0 W m^−2^ over EUR), and promotes sensible heat flux (4.7 ± 3.1 W m^−2^ over NA and 5.0 ± 3.2 W m^−2^ over EUR; Fig. 3e). In contrast, in other regions, the changes in the four types of energy fluxes are much smaller (e.g. ‒0.5 ± 0.3 W m^−2^ for latent heat flux versus ‒9.8 ± 3.2 W m^−2^ over EUR). Meanwhile, the enhanced LAC not only induces a concurrent evapotranspiration deficit and sensible heat flux increase, but also results in a significant negative correlation between these two variables (the linear correlation coefficient *r* = −0.93 and −0.94, respectively, in NA and EUR, and *p* < 0.01 for both coefficients, Fig. S17). This robust relationship demonstrates that the LAC-enhanced reduction in evapotranspiration directly contributes to amplified sensible heating through land-atmosphere feedback processes.

In conclusion, as the differences in GHG concentrations between low and high emission scenarios increase after the 2060s, the LAC effect in NA and EUR establishes a powerful positive feedback: higher land SAT enhances potential evapotranspiration (Fig. S18), desiccating the land surface; drier soils subsequently increase the proportion of incoming solar radiation converted to sensible heat; this warming further intensifies evaporative demand, thus intensifying the dryness of the land surface. Notably, this feedback remains weak in other regions where LAC is less pronounced.

### LAC effects on gross primary production

**Figure 4.**
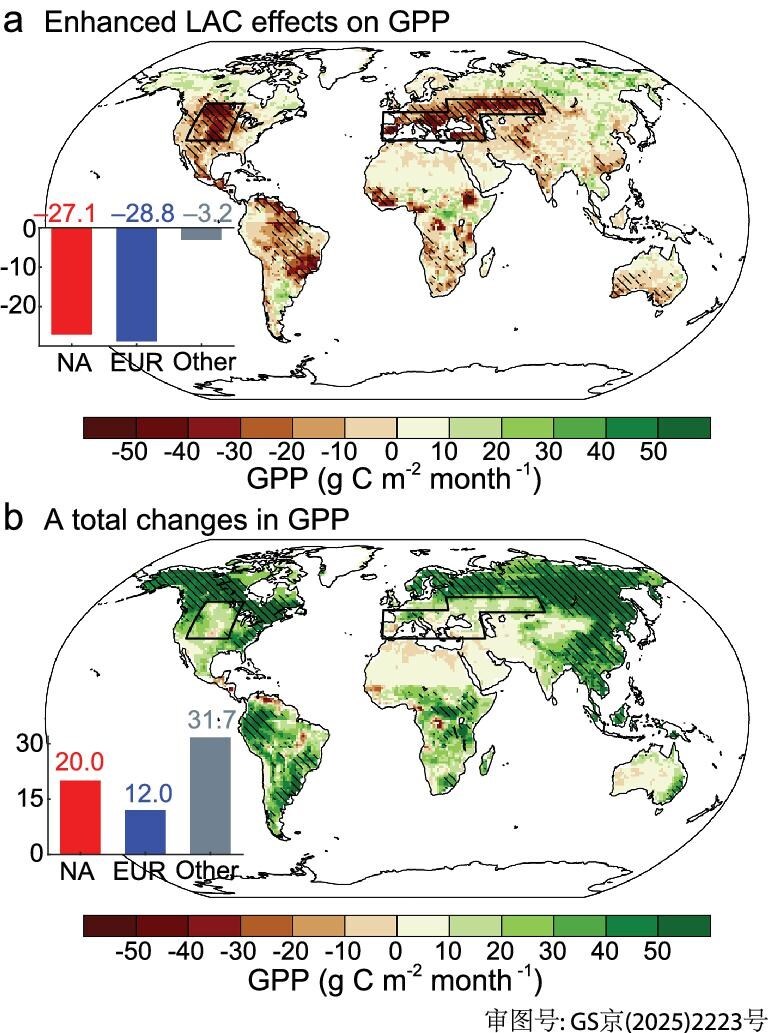
Effects of increased GHG emissions and LAC on terrestrial gross primary production (GPP). (a) Spatial distribution of the difference in LAC-caused summer GPP (g C m^−2^month^−1^) anomaly between SSP585 and SSP126 experiments during 2060‒2099. (b) Same as (a), but for the total difference (SSP585−SSP126) in summer GPP. Red, blue, and gray bars show the latitude-weighted regional averages over NA, EUR, and Other (except for desert regions, e.g. North Africa, Western Asia, and Central and Western Australia). Black lines delineate the regions of NA and EUR. In (a), areas are marked with stippling where the changes in GPP are less than the global land average in at least 80% of the models (3+/4). In (b), areas are marked with stippling where the changes in GPP exceed the global land average in at least 80% of the models (3+/4).

Terrestrial gross primary productivity (GPP) plays a crucial role in the net carbon balance of the terrestrial biosphere and in regulating atmospheric CO_2_ through its impact on the ecosystem carbon balance [42,43]. Previous studies have shown that extreme high-temperatures and droughts reduce GPP through direct physiological effects such as water limitation and heat stress and indirect effects including wildfires and insect outbreaks [44,45]. Our calculations show that strengthened LAC due to increased GHG emissions will substantially reduce GPP over NA and EUR in the late 21st century (‒27.1 ± 20.1 and ‒28.8 ± 16.9 g C m^−2^month^−1^, respectively; Fig. 4a) via increased hot and dry extremes. Such a reduction in GPP offsets the positive effect of CO_2_ fertilization on vegetation and makes GPP relatively insensitive to GHG emissions in these two regions (20.0 ± 12.0 and 12.0 ± 11.7 g C m^−2^month^−1^, respectively; Fig. 4b). In other regions (except for desert regions, e.g. North Africa, Western Asia, and Central and Western Australia), where the LAC effect is insignificant, the fully uncontrolled GHG emissions can considerably promote the carbon sequestration of vegetation (31.7 ± 11.9 g C m^−2^month^−1^). The negative impacts of LAC on GPP in NA and EUR are robust across individual models (Figs S19–S20).

## DISCUSSION AND CONCLUSIONS

This study identifies NA and EUR as the primary hotspots for warming and drying under fully uncontrolled GHG emissions, based on analyses using CMIP6 ScenarioMIP and LS3MIP experiments. Our findings highlight that enhanced land-air coupling (LAC), particularly the local drier-hotter feedback, plays a pivotal role in driving the unusual warming and drying observed in these regions. Notably, both NA and EUR are already experiencing increasingly severe heatwaves and droughts [17,30,41,46‒49]. Our results project that, without controlling GHG emissions, these regions will face more frequent and intense extreme hot and dry events, with nearly one-third of the land area undergoing significant climate degradation. Previous studies have focused primarily on LAC and its warming and drying effects [18–20,25–27,29]. Building on this foundation, we further demonstrate that increased GHG emissions intensify LAC-induced warming and drought, while quantitatively assessing their exact contributions. These findings underscore the critical role of emission mitigation in alleviating LAC-amplified climate extremes. Furthermore, deteriorating climatic conditions are likely to offset CO_2_ fertilization benefits for vegetation. Enhanced LAC poses direct and escalating threats to both regional ecological security and food production systems. NA and EUR require heightened attention to the ecological and agricultural impacts of increasing GHG emissions—particularly for key crops nearing their climatic thresholds [50–52]. Conversely, if GHG emissions are strictly curtailed globally, warming and drying in NA and EUR can be substantially reduced (Fig. S21), allowing these regions to achieve the greatest climatic benefits. Therefore, countries in North America and Europe should have stronger impetus to implement and assist other countries in adopting climate mitigation strategies, accelerating global efforts to achieve carbon peak and carbon neutrality targets.

Finally, while our findings are subject to uncertainties inherent in climate model parameterizations and projections of future radiative forcing, we have taken comprehensive measures to ensure robust conclusions. By incorporating the full ensemble of available CMIP6 models—particularly those demonstrating reliable performance in simulating historical soil moisture variability relevant to LAC—we effectively reduce random errors through multi-model mean values while quantitatively assessing inter-model variability. Importantly, although individual models exhibit some differences in the magnitude of projected changes, they consistently reproduce the key spatial patterns and relative differences between regions. This remarkable agreement across all models, as evidenced by our single-model analyses, provides strong confidence in the robustness of our principal findings regarding the amplified dry-hot response in NA and EUR under climate change.

## MATERIALS AND METHODS

### CMIP6 models

This study used the LS3MIP and ScenarioMIP experiments in CMIP6 global climate models to analyze the effects of increased emissions and LAC on climate change. The ScenarioMIP reflects future climate change under different SSPs. We selected SSP1-2.6 and SSP5-8.5—corresponding to two future scenarios: GHG emissions are strictly controlled (low-emission scenario, SSP1-2.6) and GHG emissions are fully uncontrolled (high-emission scenario, SSP5-8.5)—to analyze the effects of different emission scenarios on climate change. Twenty-four global climate models (Table S1) in ScenarioMIP are used in this study.

The LS3MIP reflects the influence of land surface processes on climate, and the LFMIP-pdLC experiment in LS3MIP was used here to analyze the effects of LAC on climate change. In the LFMIP-pdLC experiment, while soil moisture was fixed to the climatological state of the historical period (1980–2014), all other components, including LAC processes and ocean-atmosphere interactions remained identical to the SSP experiments. Therefore, the difference between SSP and LFMIP-pdLC experiments effectively isolates the LAC climate effect. This methodology has been widely adopted in studies investigating LAC impacts on weather and climate [18,20–27,29]. The LAC effect was isolated by using the difference between the fully coupled experiment (scenario experiments SSP126 and SSP585) and the fixed soil moisture experiment (LFMIP-pdLC). In the LFMIP-pdLC experiment, the r1i1p1f1 member corresponded to the low-emission scenario (pdLC126), and the r1i1p1f2 member corresponded to the high-emission scenario (pdLC585). The LFMIP-pdLC experiments from six CMIP6 global climate models: CESM2, CMCC-ESM2, EC-Earth3, IPSL-CM6A-LR, MIROC6, and MPI-ESM1-2-LR. The EC-Earth3 and MIROC6 models lack output of the GPP element; the analysis with GPP comes from the other four models (CESM2, CMCC-ESM2, IPSL-CM6A-LR, and MPI-ESM1-2-LR). At the same time, the MIROC6 model lacks the output of latent heat flux; the analysis about latent heat flux comes from the other five models (CESM2, CMCC-ESM2, EC-Earth3, IPSL-CM6A-LR, and MPI-ESM1-2-LR). Experiments in CMIP6 (1850–2014) simulates the historical climate with all external forcings in order to assess the model’s ability to simulate climate change.

Daily maximum SAT data were used to analyze extreme high-temperature (Fig. 1b, Figs S6 and S7), and the remaining analysis was based on monthly meteorological elements. Because some models did not provide corresponding daily maximum SAT data, only 19 models were involved in the analysis of extreme high-temperature (Table S1). The surface soil moisture (10 cm) is analyzed in this study, and the relevant results about deep root zone soil (100 cm) are supplied in the Supplementary Information (Fig. S16). Because the LAC effect is most intense in summer, the extra-tropical summer was chosen for this study (i.e. June‒August in the Northern Hemisphere, and December‒February in the Southern Hemisphere).

To reduce the influence of inter-model uncertainty, the results herein are reported as multi-model mean values, and the standard deviation of the results of different models in quantitative analysis is used to represent the uncertainty among models. Meanwhile, we examined the results from individual models, and most of the models’ results showed similar conclusions, verifying the robustness of our research results (Figs S4–S9, S12–S15, S19 and S20). For the CMIP6 climate models, previous studies have demonstrated that most models successfully reproduce both the climatological patterns and temporal variability of historical soil moisture and evapotranspiration in most regions, except for some high-latitude, high-altitude, and extremely arid regions [53]. Meanwhile, relevant researches also indicate that NA and EUR are hotspots of LAC during the historical period [3,18,23,25]. These researches confirm that the CMIP6 models perform well in simulating soil moisture and LAC in NA and EUR, thereby ensuring the reliability of future projections.

In Supplementary Information, we compare SAT and aridity index in the future under the high-emission scenario (2060–2099, in the SSP5-8.5 experiment) with modern times (1975–2014, in the historical experiment) and find that the future will indeed become drier and hotter in NA and EUR compared to other regions (Figs S22 and S23). The historical experiment in CMIP6 (1850–2014) simulates the historical climate with all external forcings in order to assess the model’s ability to simulate climate change.

### Aridity index

We used the ratio of potential evapotranspiration to precipitation as an aridity index to characterize the degree of dryness or wetness in a region and to depict regional climate change in terms of aridity (Fig. 2f, Figs S2, S10), where humid = aridity index < 1; semi-humid = 1 ≤ aridity index < 1.5; semi-arid = 1.5 ≤ aridity index < 4; arid = 4 ≤ aridity index < 10; and extremely arid = aridity index ≥ 10 [37,38]. Because most models do not provide potential evapotranspiration, we used the Penman‒Monteith algorithm to calculate potential evapotranspiration; this is considered the most reliable method for calculating potential evapotranspiration and aridity [54,55]:


(1) \begin{eqnarray*}
{E}_{TO} = \displaystyle\frac{{0.408\Delta \left( {{R}_n - G} \right) + \gamma \displaystyle\frac{{900}}{{T + 273}}{U}_2\left( {{e}_s - {e}_d} \right)}}{{\Delta + \gamma \left( {1 + 0.34{U}_2} \right)}},
\end{eqnarray*}

where ${E}_{TO}$ is the potential evapotranspiration, $\Delta $ is the slope of the vapor pressure‒temperature curve (kPa °C^−1^), ${R}_n$ is the net radiation at the surface (MJ m^−2^ day^−1^), *G* is the soil heat flux density at the soil surface (MJ m^−2^ day^−1^),$\ \gamma $ is the psychometric constant (kPa °C^−1^), *T* is the mean monthly SAT at 2 m height (°C),$\ {U}_2$ is the wind speed at 2 m height (m s^−1^), ${e}_s$ is the saturation vapor pressure at 2 m height (kPa), and ${e}_d$ is the actual vapor pressure at 2 m height (kPa).

We calculated ${e}_s$ and ${e}_d$ pressure using SAT and relative humidity (${R}_H$):


(2) \begin{eqnarray*}
{e}_s = 0.6108exp\left( {\displaystyle\frac{{17.27T}}{{T + 237.3}}} \right),
\end{eqnarray*}

(3) \begin{eqnarray*}
{e}_d = {e}_s{R}_H.
\end{eqnarray*}

Because the wind speed given by the models was measured at 10 m, it was necessary to convert it to 2 m wind speed:


(4) \begin{eqnarray*}
{U}_2 = {U}_Z\displaystyle\frac{{4.87}}{{\ln \left( {67.8 \times Z - 5.42} \right)}},
\end{eqnarray*}

where *Z* is the height of wind speed.

$\gamma $ was calculated as follows:


(5) \begin{eqnarray*}
\gamma = \displaystyle\frac{{{c}_pP}}{{\varepsilon \lambda }} = 0.665 \times {10}^{ - 3}P,
\end{eqnarray*}

where ${c}_p$ is the specific heat at constant pressure (1.013 × 10^−3^ MJ kg^−1^ °C^−1^), *P* is the surface air pressure (kPa), $\varepsilon $ is the ratio of water vapor to dry air (0.622), and$\ \lambda $ is the latent heat of vaporization (2.45 MJ kg^−1^).

For the long-term climate state, because the soil heat flux is small, *G* was ignored when calculating the aridity index.

$\Delta $ was calculated as follows:


(6) \begin{eqnarray*}
{\mathrm{\Delta }} = \displaystyle\frac{{4098 \times {e}_d}}{{{{\left( {T + 237.3} \right)}}^2}}.
\end{eqnarray*}

${R}_n$ was calculated as follows:


(7) \begin{eqnarray*}
{R}_n = {L}_ \downarrow + {S}_ \downarrow - {L}_ \uparrow - {S}_ \uparrow ,
\end{eqnarray*}

where ${L}_ \downarrow $ and ${L}_ \uparrow $ represent downward and upward long-wave radiation, respectively (W m^−2^), and ${S}_ \downarrow $ and ${S}_ \uparrow $ represent downward and upward short-wave radiation, respectively (W m^−2^).

When calculating the effect of emissions on the aridity index, BCC-CSM2-MR, NESM3, and TaiESM1 lack surface relative humidity values. Therefore, Fig. 1c depicts multi-model mean results of the other 21 models. In Fig. S23, to compare the degree of change in the aridity index between the historical period and late 21st century, we used the historical and SSP5-8.5 experiments in CMIP6. Because four models (AWI-CM-1–1-MR, BCC-CSM2-MR, NESM3, and TaiESM1) in the historical experiment were missing some elements, Fig. S23 depicts multi-model mean results of the other 20 models.

### Extreme high-temperature

Daily data from CMIP6 models were used to analyze the impact of increased emissions on extreme high-temperature. Through counting the 90% of the maximum extra-tropical summer SAT for the period 2060‒2099 under high- and low-emission scenarios, the difference between these values was then used to represent the impact of increased emissions on extreme high-temperature intensity in 2060‒2099.

### EEMD method

We employed the Ensemble Empirical Mode Decomposition (EEMD) method to eliminate interannual variations of time series, enabling better observation and analysis of the long-term impacts of GHG and LAC effects on temperature and drought. The EEMD method’s advantage lies in its time series decomposition not relying on predetermined functions, but rather utilizing a self-adaptive filtering approach derived from the data itself. By introducing controlled white noise to the original data to simulate multiple observational scenarios and performing repeated computations, the final results are obtained through ensemble averaging [56].

## Supplementary Material

nwaf435_Supplemental_File

## Data Availability

All data used in this study are freely available online. The CMIP6 model simulations are from https://esgf-node.llnl.gov/search/cmip6/.
