# Supplementary material for: Extreme dry-hot in North America and Europe: the amplified role of warming-enhanced land-air coupling
Source: Natl Sci Rev. 2025 Oct 13;12(11):nwaf435. doi: 10.1093/nsr/nwaf435 (PMC12648562; doi:10.1093/nsr/nwaf435)
Supplement: nwaf435_Supplemental_File [file nwaf435_supplemental_file.docx]

Supplementary Materials for

**Extreme Dry-Hot in North America and Europe: The Amplified Role of Warming-Enhanced Land-Air Coupling**

Liang Qiao^1,2^, Zhiyan Zuo^2,3*^, Renhe Zhang^2*^, Wei Mei^2^, Deliang Chen^4,5^, Meiyu Chang^2^, Kaiwen Zhang^2^

**Supplementary table and figures**

**Table S1. List of the 24 global climate models used in this study from ScenarioMIP in CMIP6.**

| Model name | latitude grid points | longitude grid points | Monthly data | Daily data |
| --- | --- | --- | --- | --- |
| ACCESS-CM2 | 144 | 192 | √ | √ |
| AWI-CM-1-1-MR | 192 | 384 | √ | √ |
| BCC-CSM2-MR | 160 | 320 | √ | √ |
| CESM2 | 192 | 288 | √ | √ |
| CESM2-WACCM | 192 | 288 | √ |  |
| CMCC-CM2-SR5 | 192 | 288 | √ |  |
| CMCC-ESM2 | 192 | 288 | √ | √ |
| EC-Earth3 | 256 | 512 | √ | √ |
| EC-Earth3-Veg-LR | 160 | 320 | √ | √ |
| EC-Earth3-Veg | 256 | 512 | √ | √ |
| FIO-ESM-2-0 | 192 | 288 | √ |  |
| GFDL-ESM4 | 180 | 288 | √ |  |
| INM-CM4-8 | 120 | 180 | √ | √ |
| INM-CM5-0 | 120 | 180 | √ | √ |
| IPSL-CM6A-LR | 143 | 144 | √ | √ |
| KACE-1-0-G | 144 | 192 | √ | √ |
| MIROC6 | 128 | 256 | √ | √ |
| MPI-ESM1-2-HR | 192 | 384 | √ | √ |
| MPI-ESM1-2-LR | 96 | 192 | √ | √ |
| MRI-ESM2-0 | 160 | 320 | √ | √ |
| NESM3 | 96 | 192 | √ | √ |
| NorESM2-LM | 96 | 144 | √ | √ |
| NorESM2-MM | 192 | 288 | √ |  |
| TaiESM1 | 192 | 288 | √ | √ |


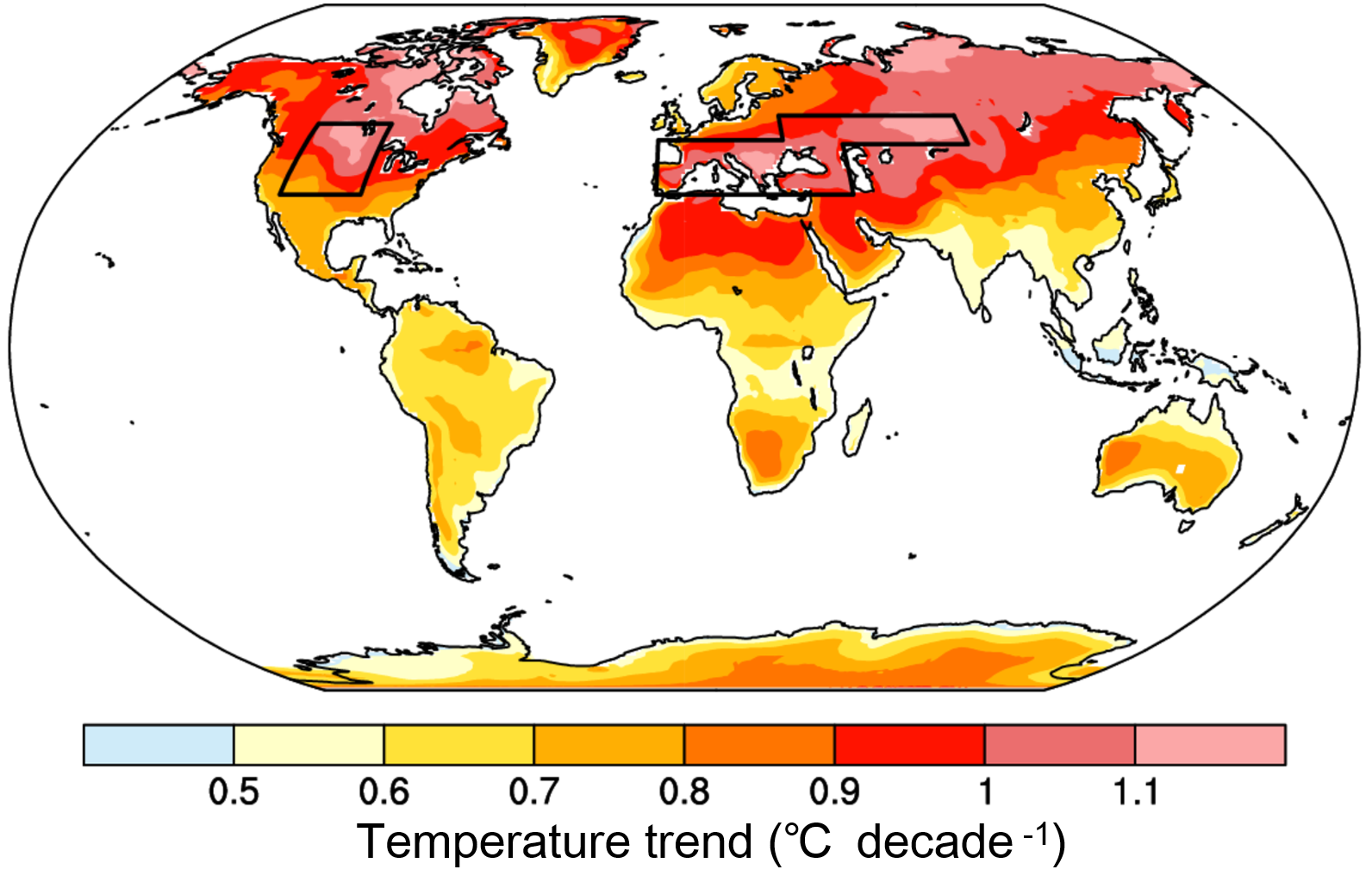


**Fig. S1. Spatial distribution of the summer surface air temperature trend (°C decade^-1^) difference between high- and low-emission scenarios (SSP5-8.5 and SSP1-2.6, respectively) for 2060‒2099.**


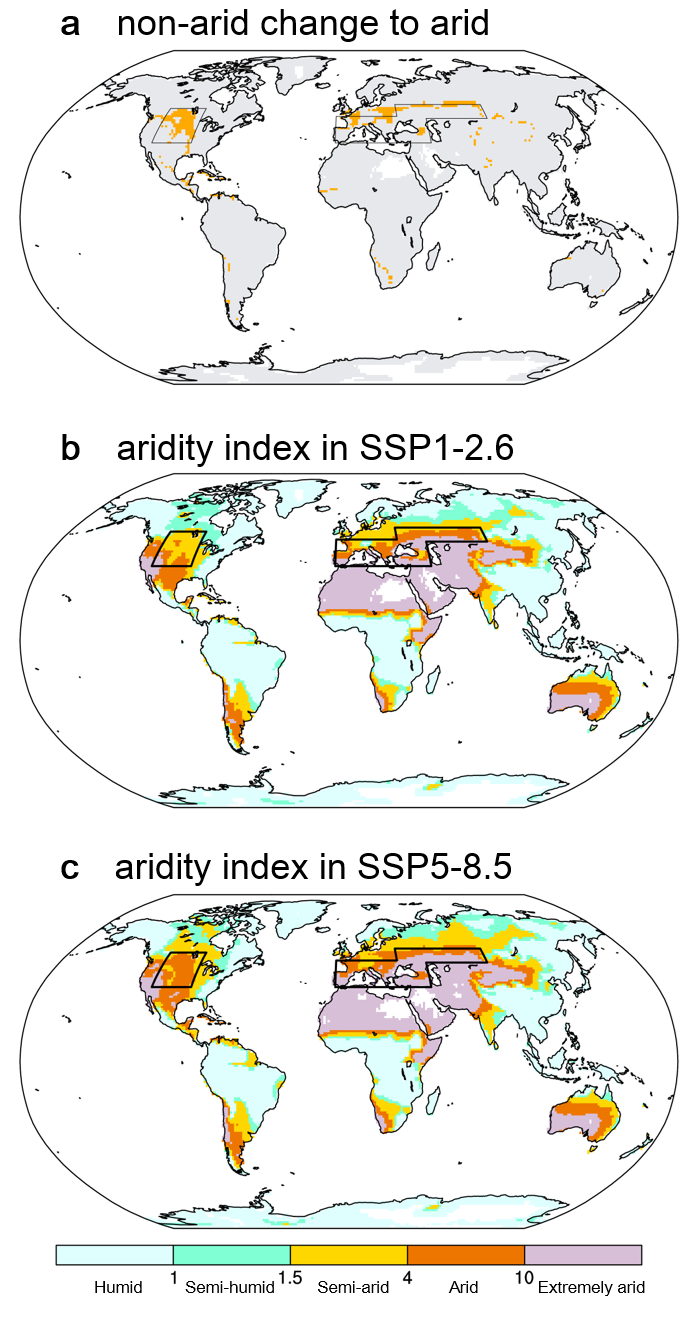


**Fig. S2. Spatial distributions of the aridity index. a**, Regions experiencing a shift within 2060‒2099 from non-arid climate condition to arid condition (orange shading in the map), obtained by a comparison between the low- and high-emission scenarios (SSP1-2.6 and SSP5-8.5). **b**, SSP1-2.6 experiment for 2060‒2099. **c**, SSP5-8.5 experiment for 2060‒2099. The climate models are consistent with Figure 1c (21 models).


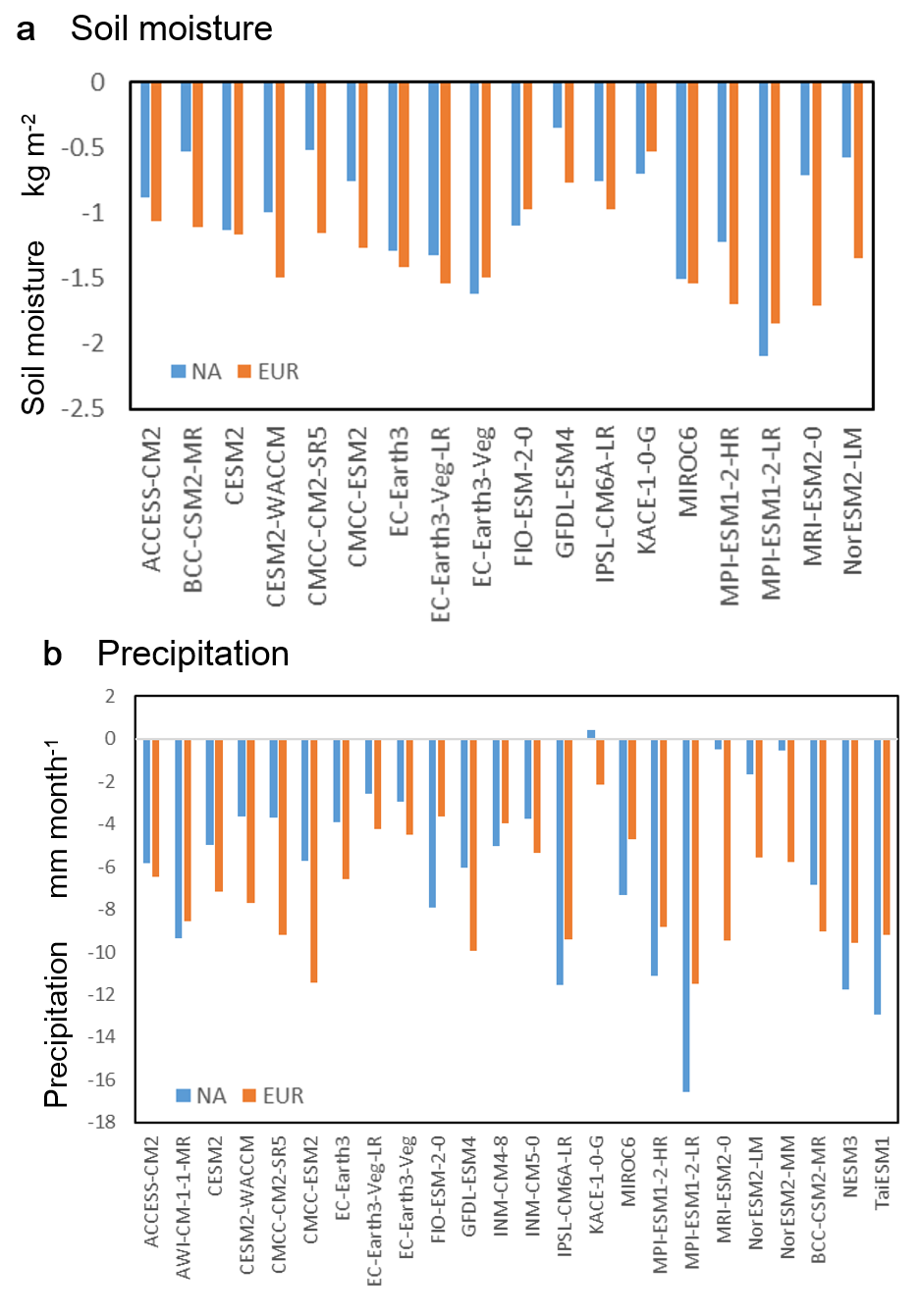


**Fig. S3. Effect of increased GHG emissions (difference between high- and low-emission scenarios) on soil moisture (kg m^-2^) and precipitation (mm month^-1^) over NA and EUR for 2060‒2099.**


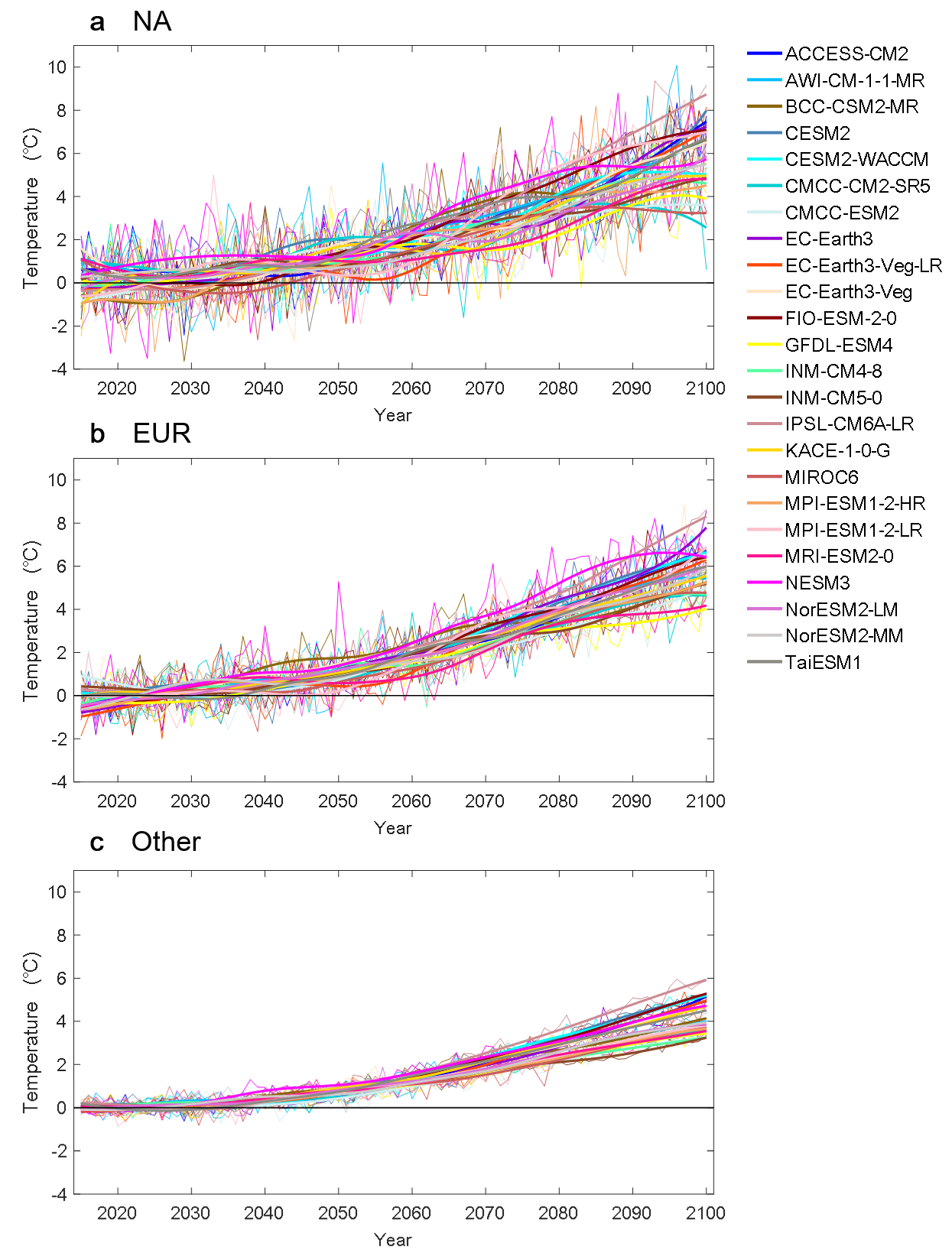


**Fig. S4. Temporal evolution of summer surface air temperature (°C) difference between high- and low-emission scenarios (SSP5-8.5 and SSP1-2.6, respectively) for 2015‒2099 in different single model. a-c** refer to North America (NA), Europe (EUR), and other regions (Other) respectively.


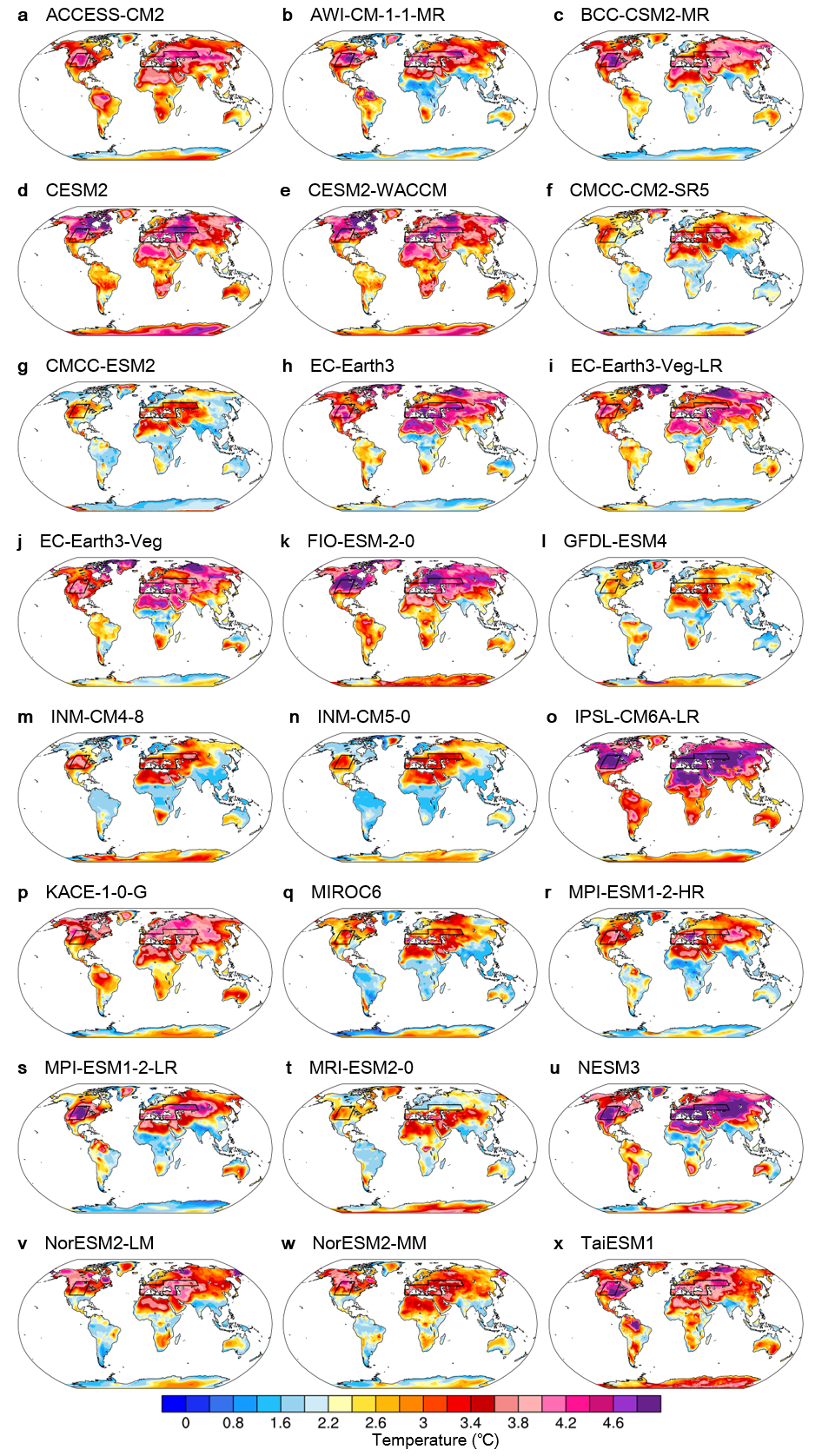


**Fig. S5.** **Spatial distribution of summer surface air temperature (°C) difference between high- and low-emission scenarios (SSP5-8.5 and SSP1-2.6, respectively) for 2060‒2099 in different single model.**


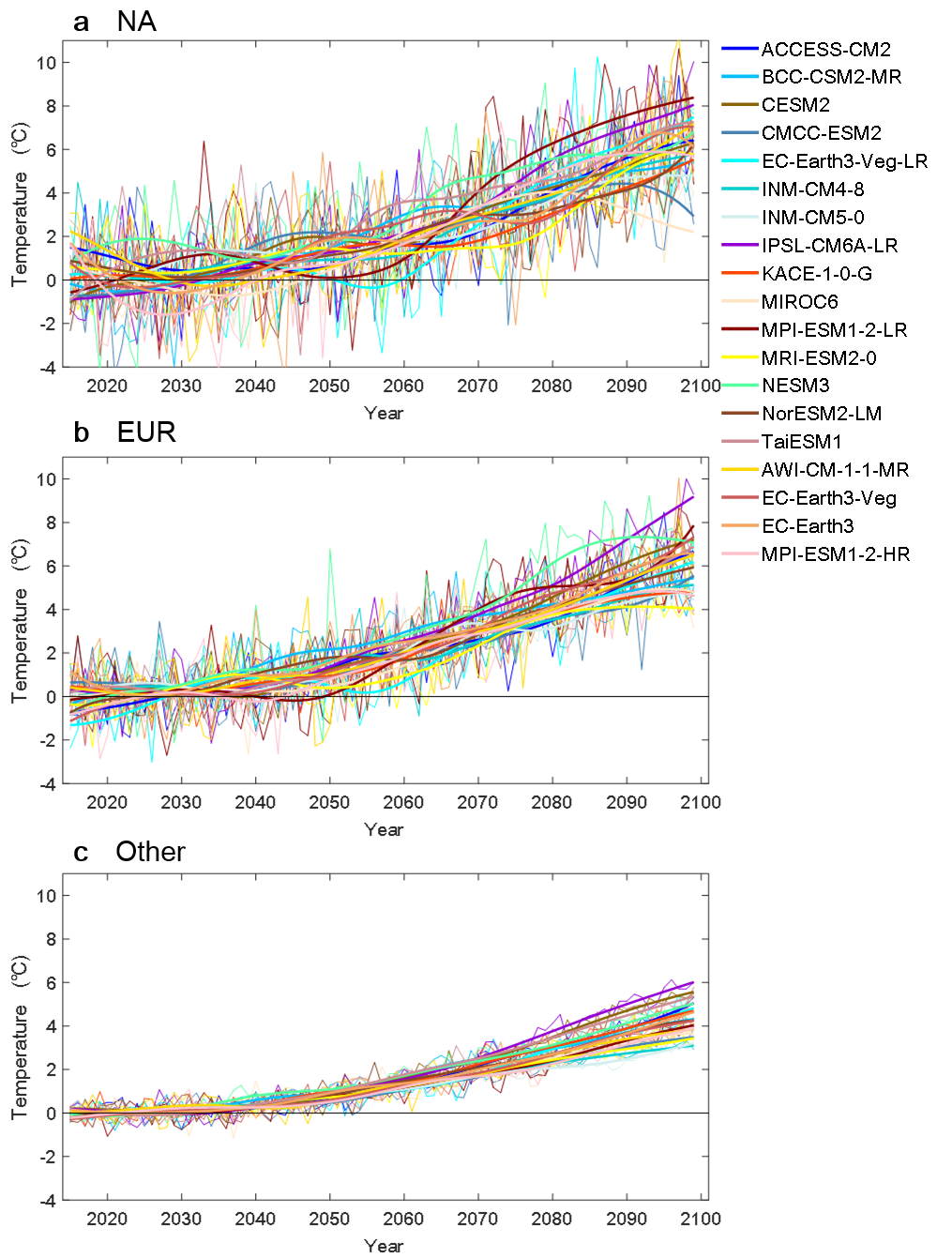


**Fig. S6. Same as Fig. S4, but for extremely high-temperature (°C)**.


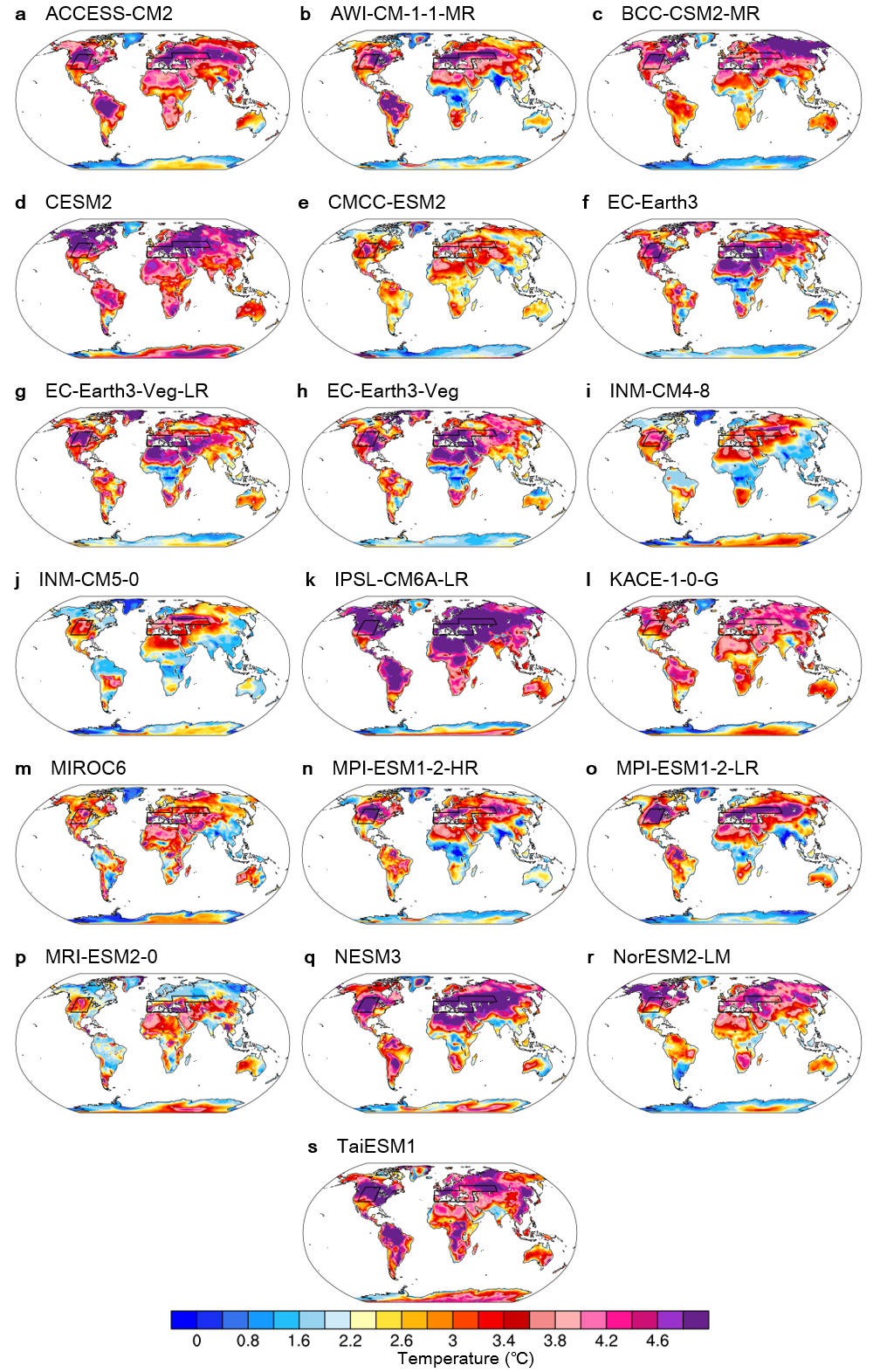


**Fig. S7. Same as Fig. 5, but for extremely high-temperature (°C).**


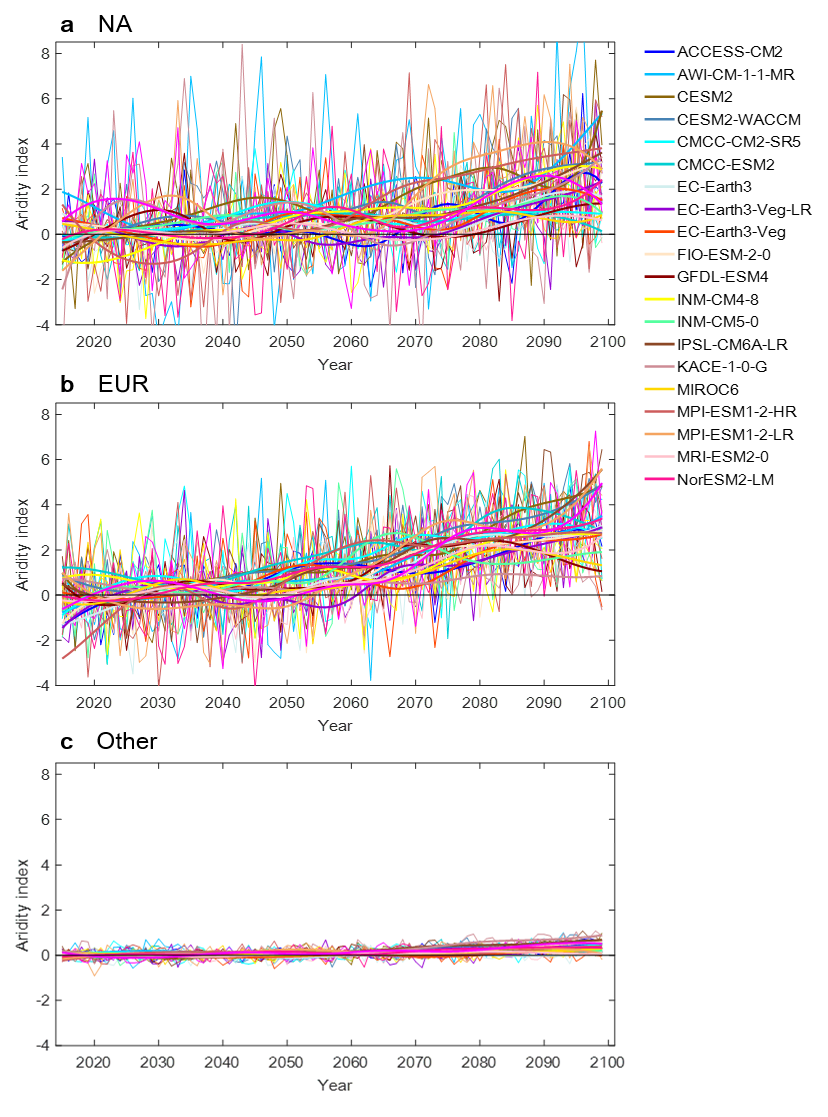


**Fig. S8. Same as Fig. S4, but for aridity index.**


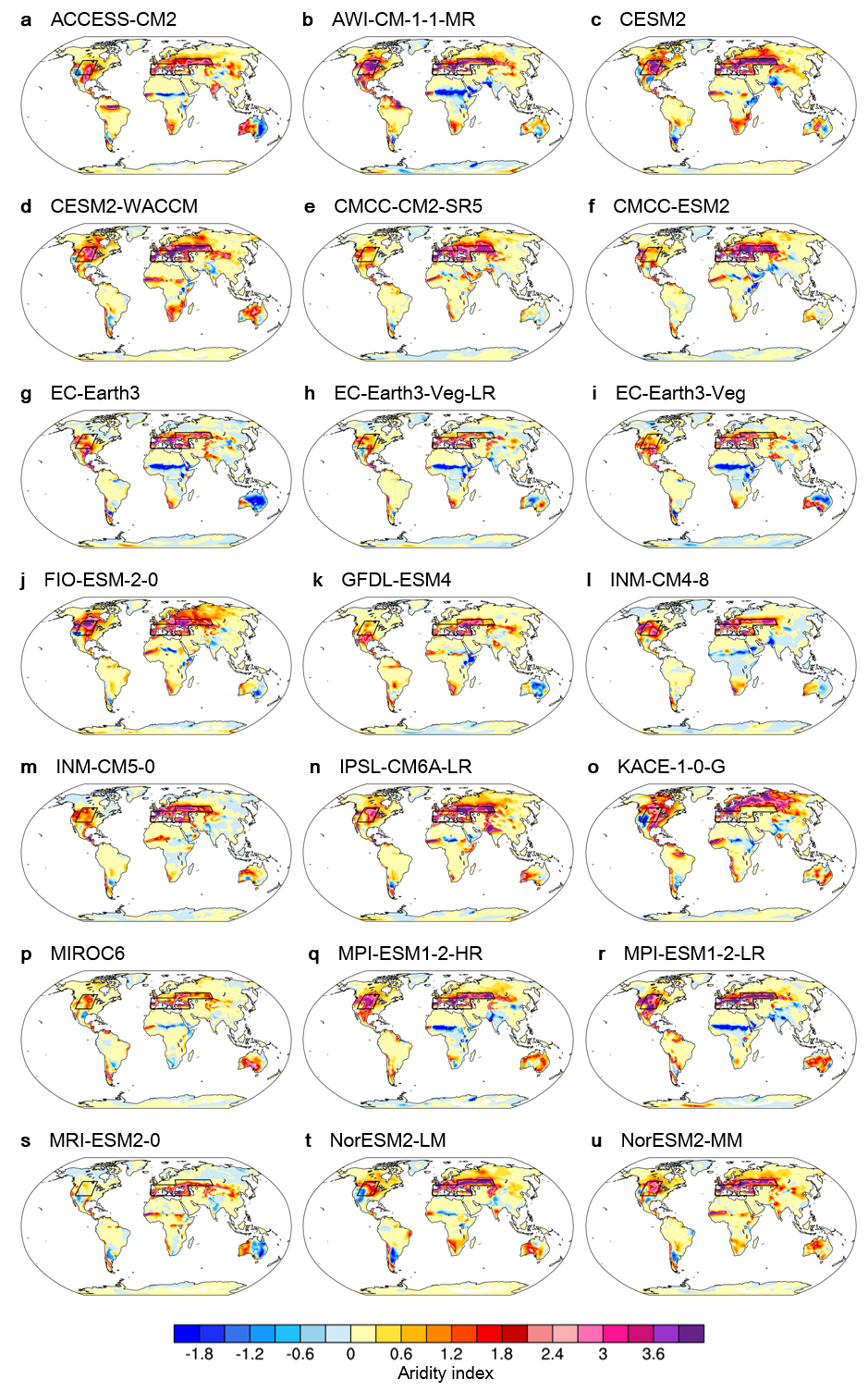


**Fig. S9. Same as Fig. S5, but for aridity index.**


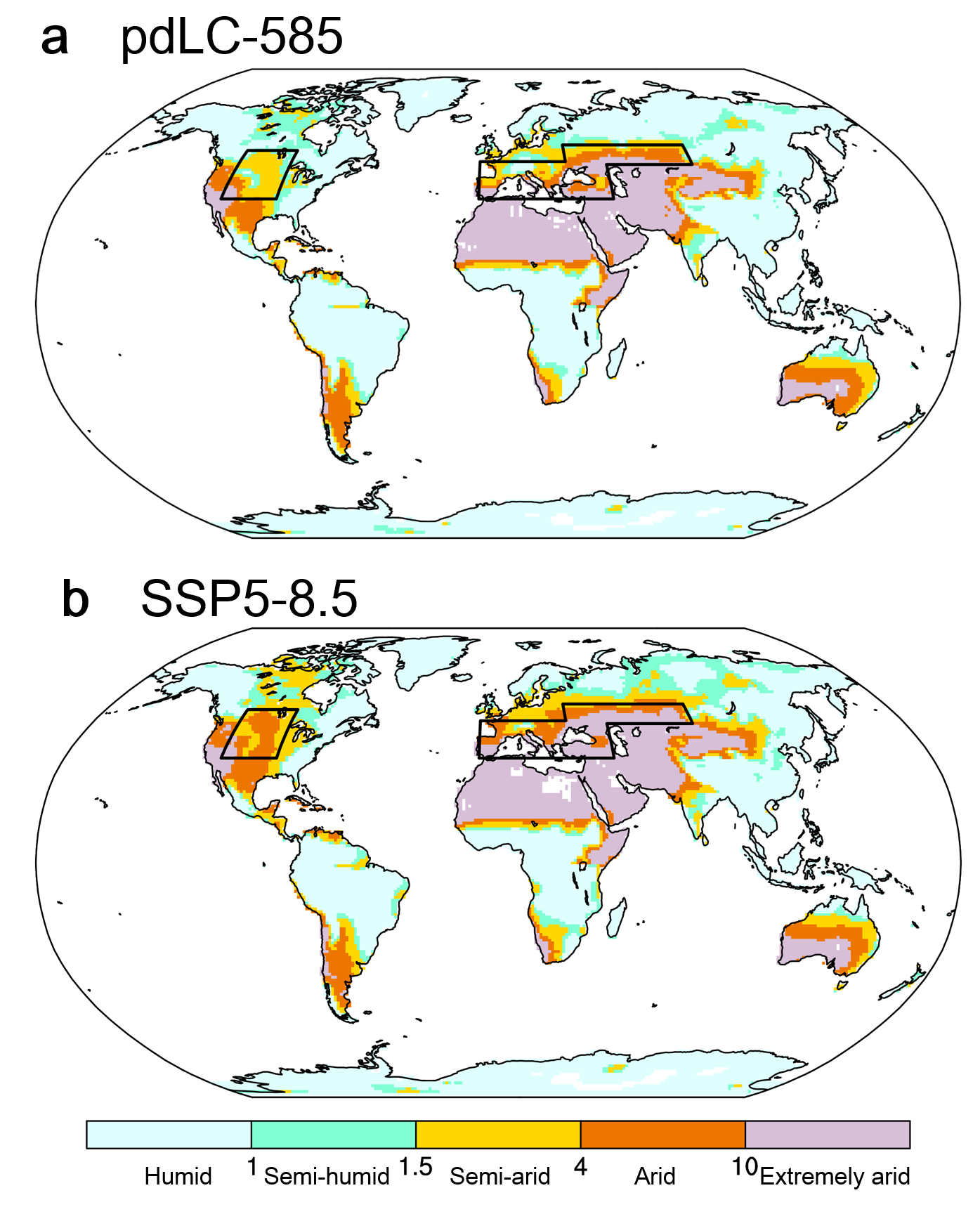


**Fig. S10. Spatial distributions of the aridity index. a**, pdLC585 experiment for 2060‒2099. **b**, SSP5-8.5 experiment for 2060‒2099. The climate models are consistent with Figure 2e (6 models).


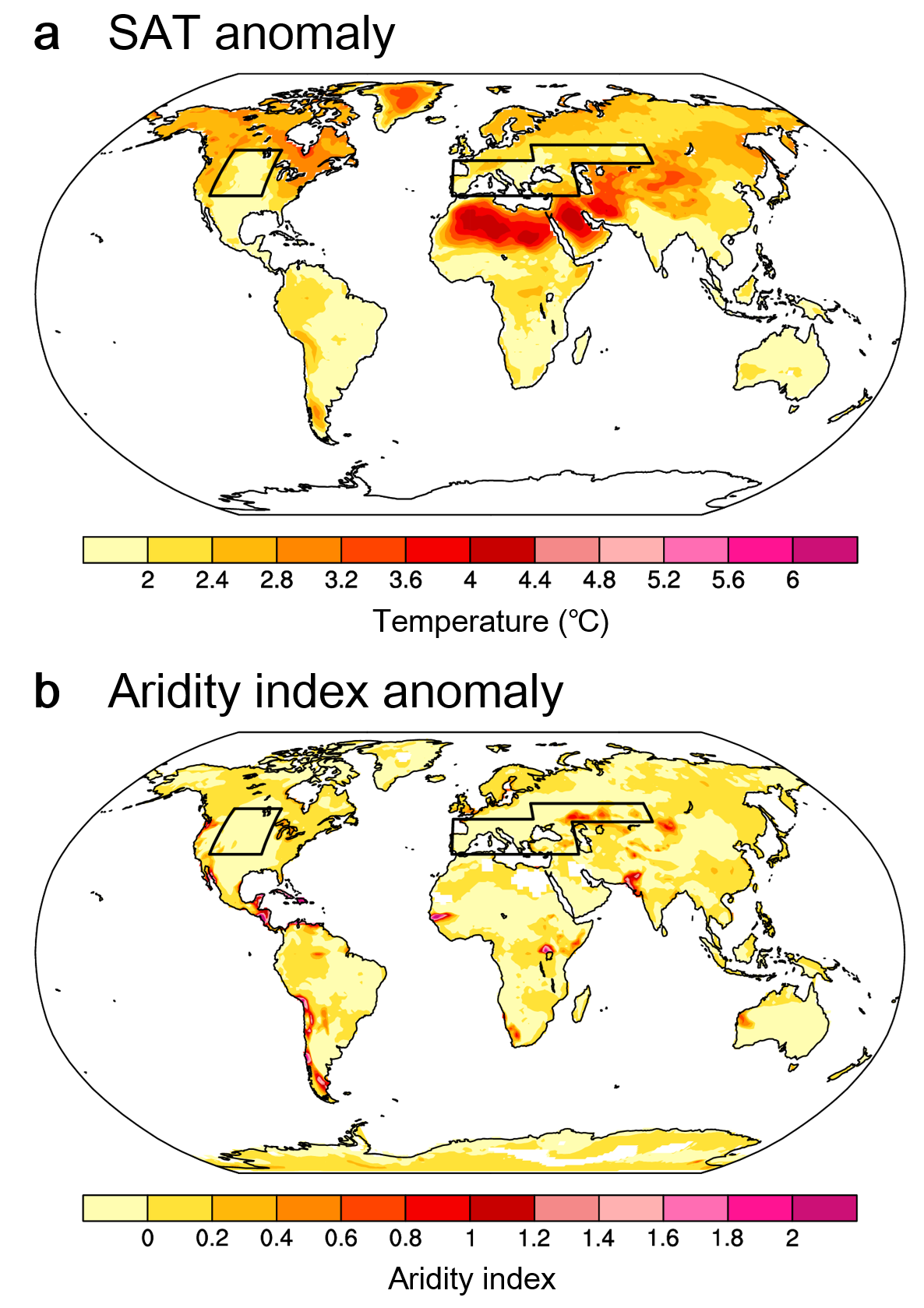


**Fig. S11. Spatial distribution of surface air temperature (SAT, ℃) and drying (aridity index) after removing the land‒air coupling effect under the high-emission scenario for 2060‒2099 ((SSP585－SSP126)－(SSP585－pdLC585) = pdLC585－SSP126). a** is SAT, and **b** is aridity index.


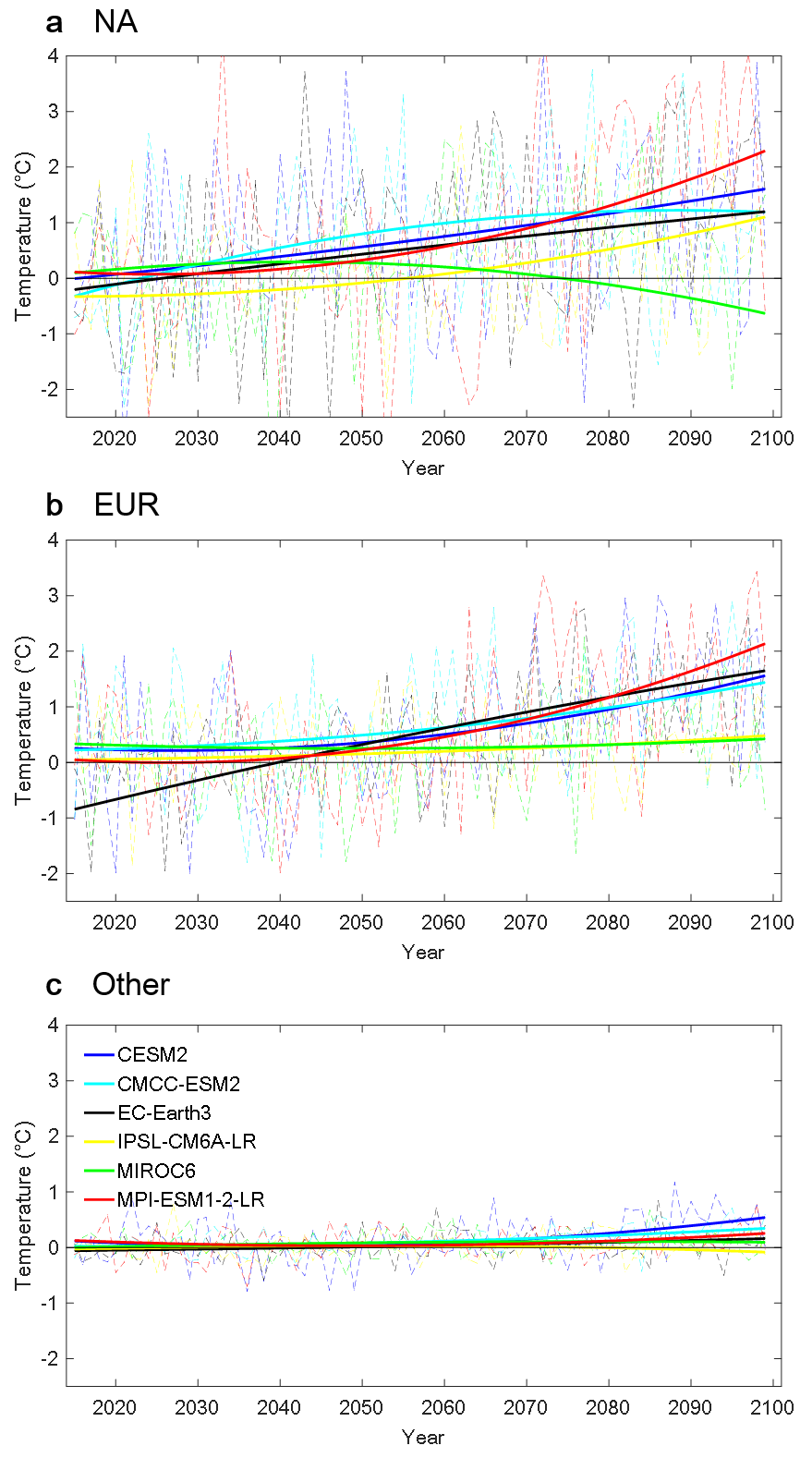


**Fig. S12. Same as Fig. 2a, but for different single model.**


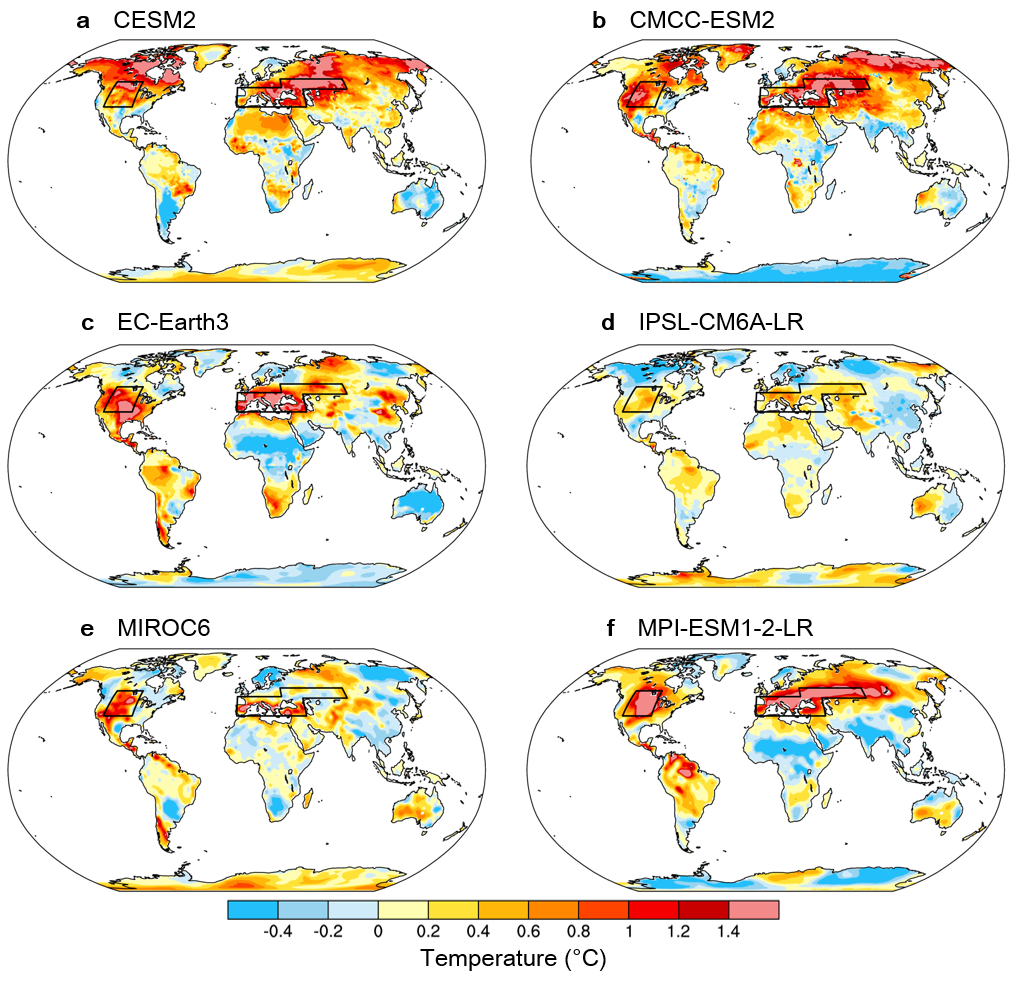


**Fig. S13. Same as Fig. 2b, but for different single model.**


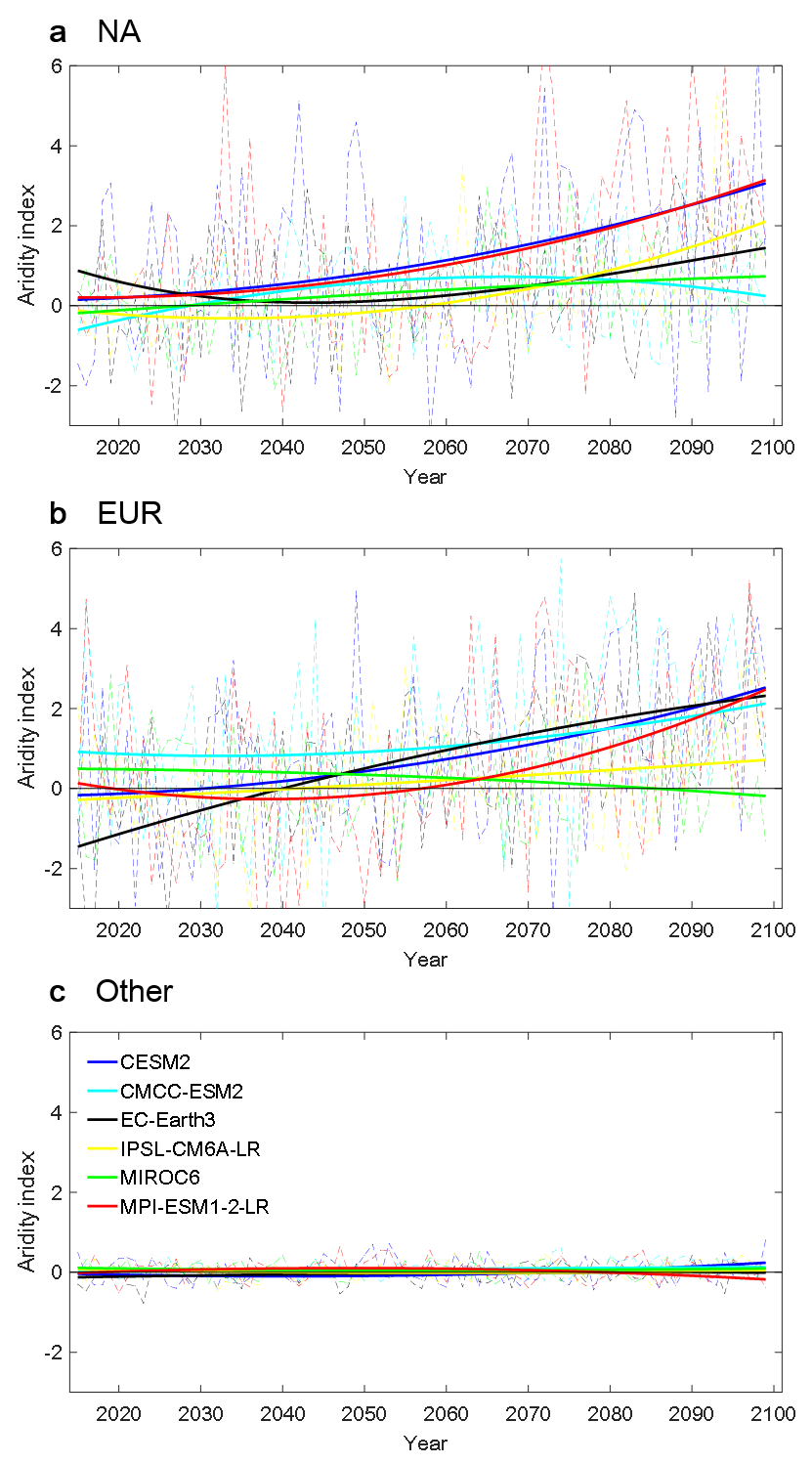


**Fig. S14. Same as Fig. 2d, but for different single model.**


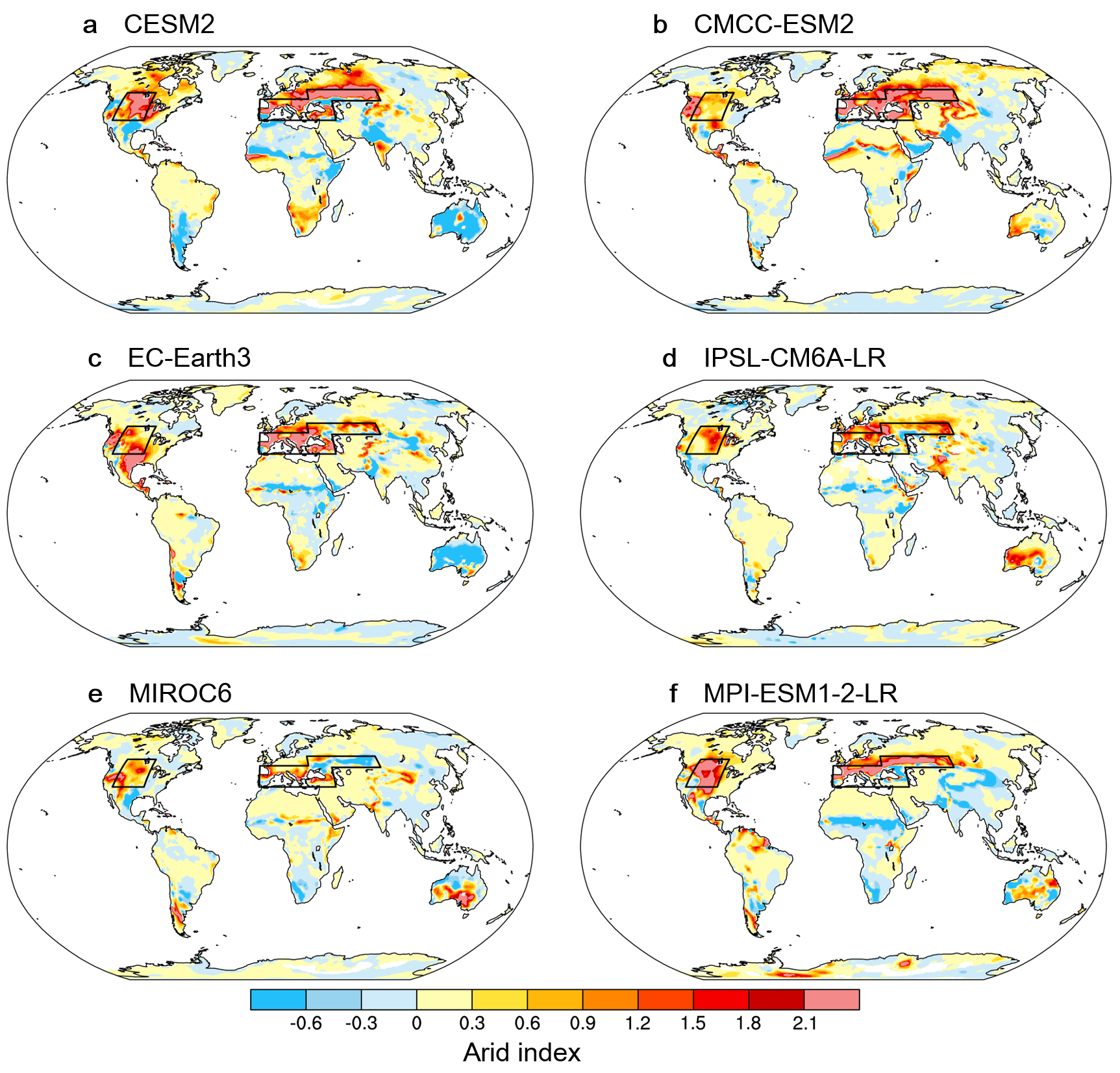


**Fig. S15. Same as Fig. 2e, but for different single model.**


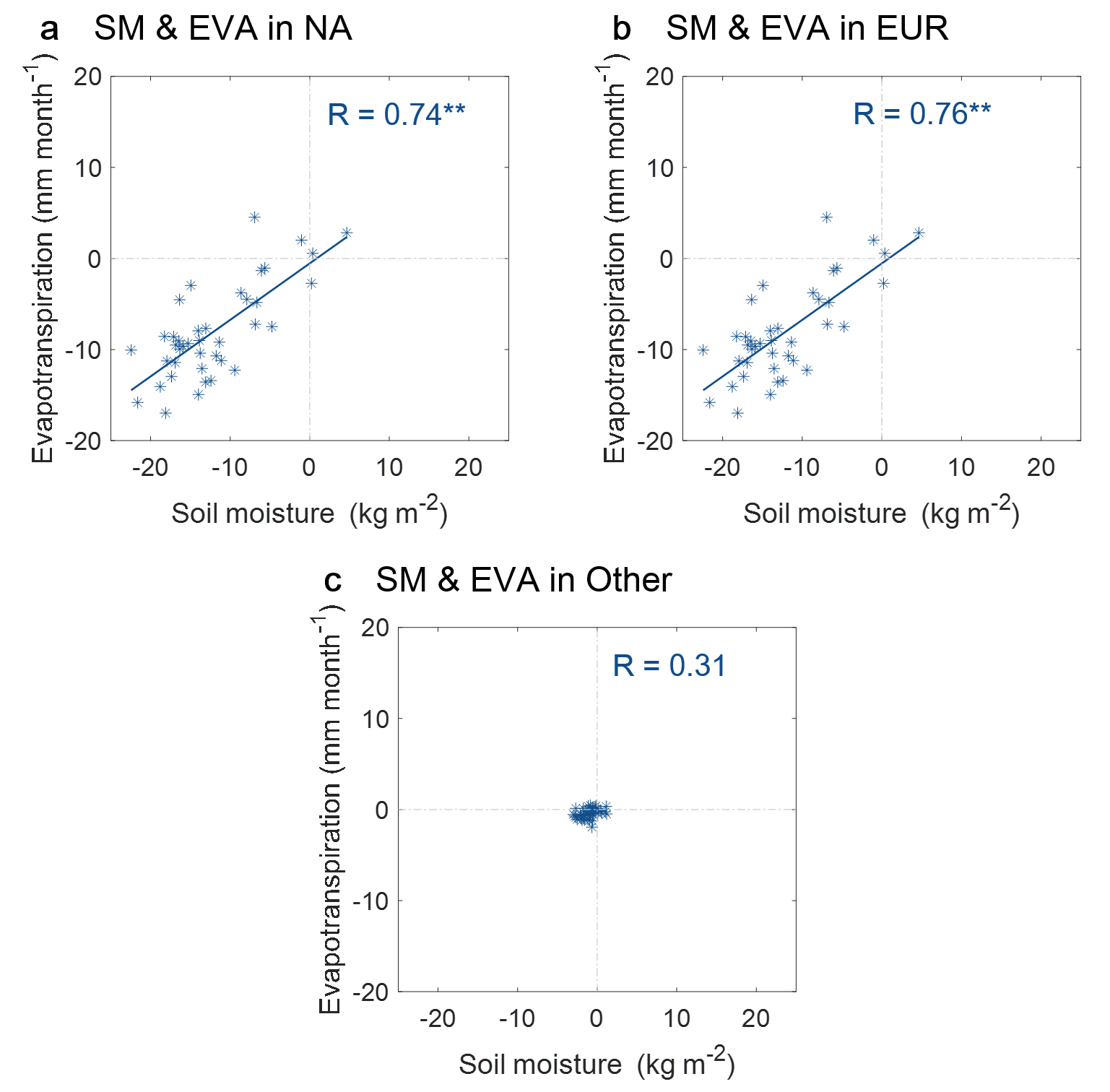


**Fig. S16. Same as Figs. 3 a‒c, but soil moisture (SM) values (kg m^−2^) come from the** **deep soil layer (100 cm, root zone soil moisture).**


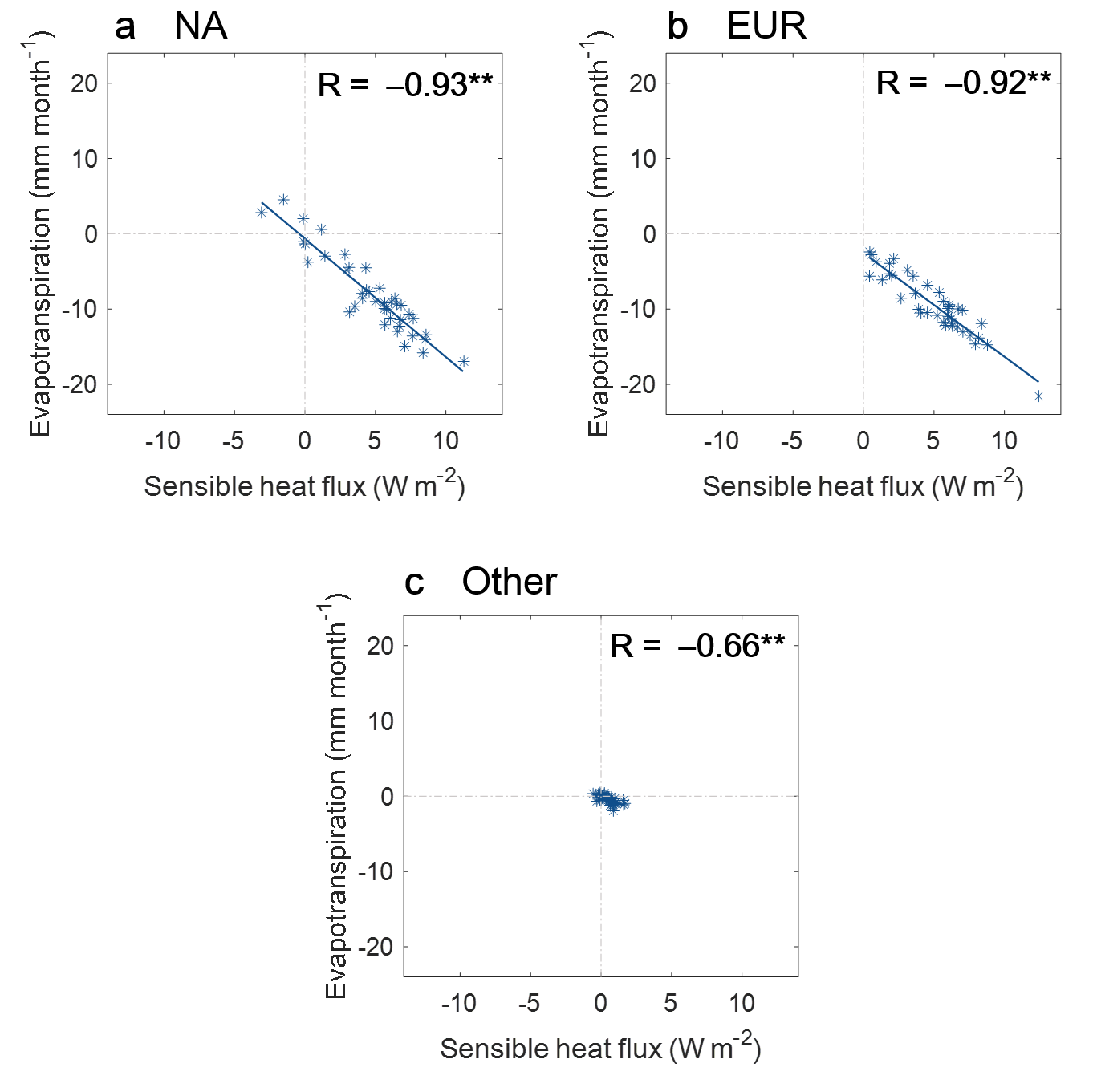


**Fig. S17. Scatter plot of surface evapotranspiration anomaly (mm month^-1^) due to enhanced LAC versus surface sensible heat flux anomaly (W m^-2^) due to enhanced LAC over NA (a), EUR (b), and Other (c) for 2060‒2099.** The correlation coefficient between evapotranspiration and sensible heat flux is shown in the top right, with ‘**’ indicating that the correlation coefficient is statistically significant at a 0.01 level.


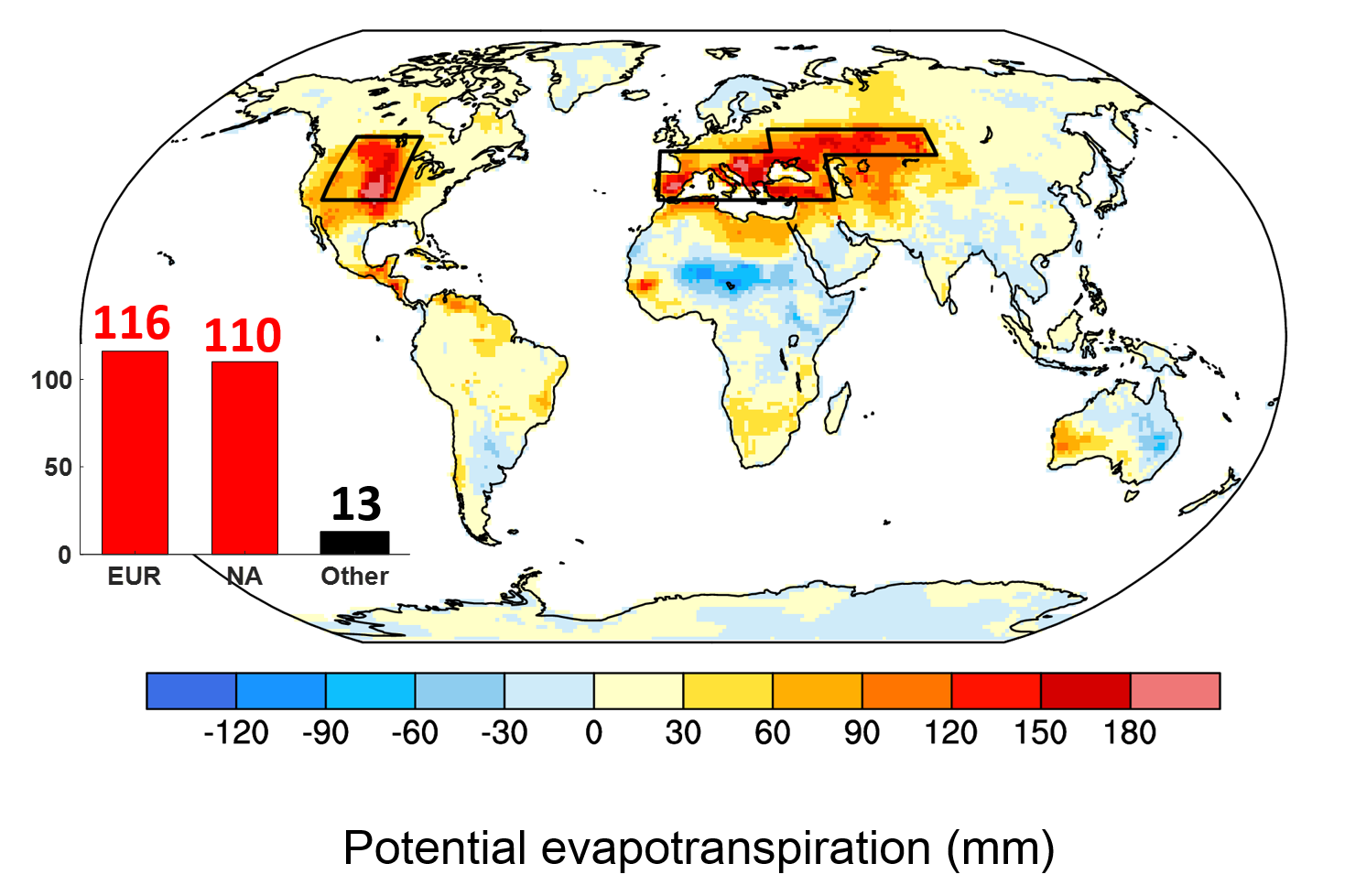


**Fig. S18.** **Same as Figs. 2b,e, but for potential evapotranspiration** **(mm month^-1^).**


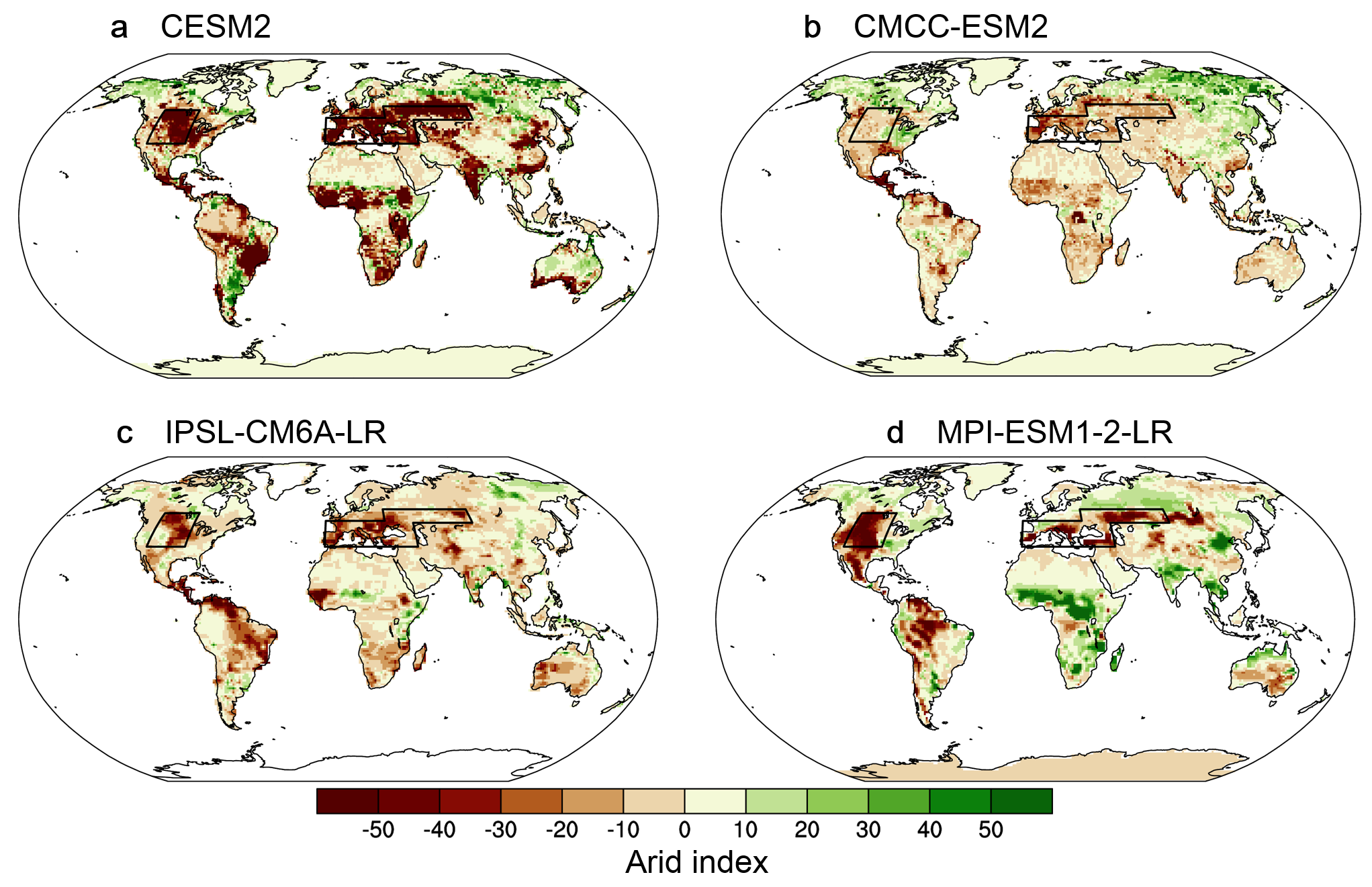


**Fig. S19. Same as Fig. 4a, but for different single model.**


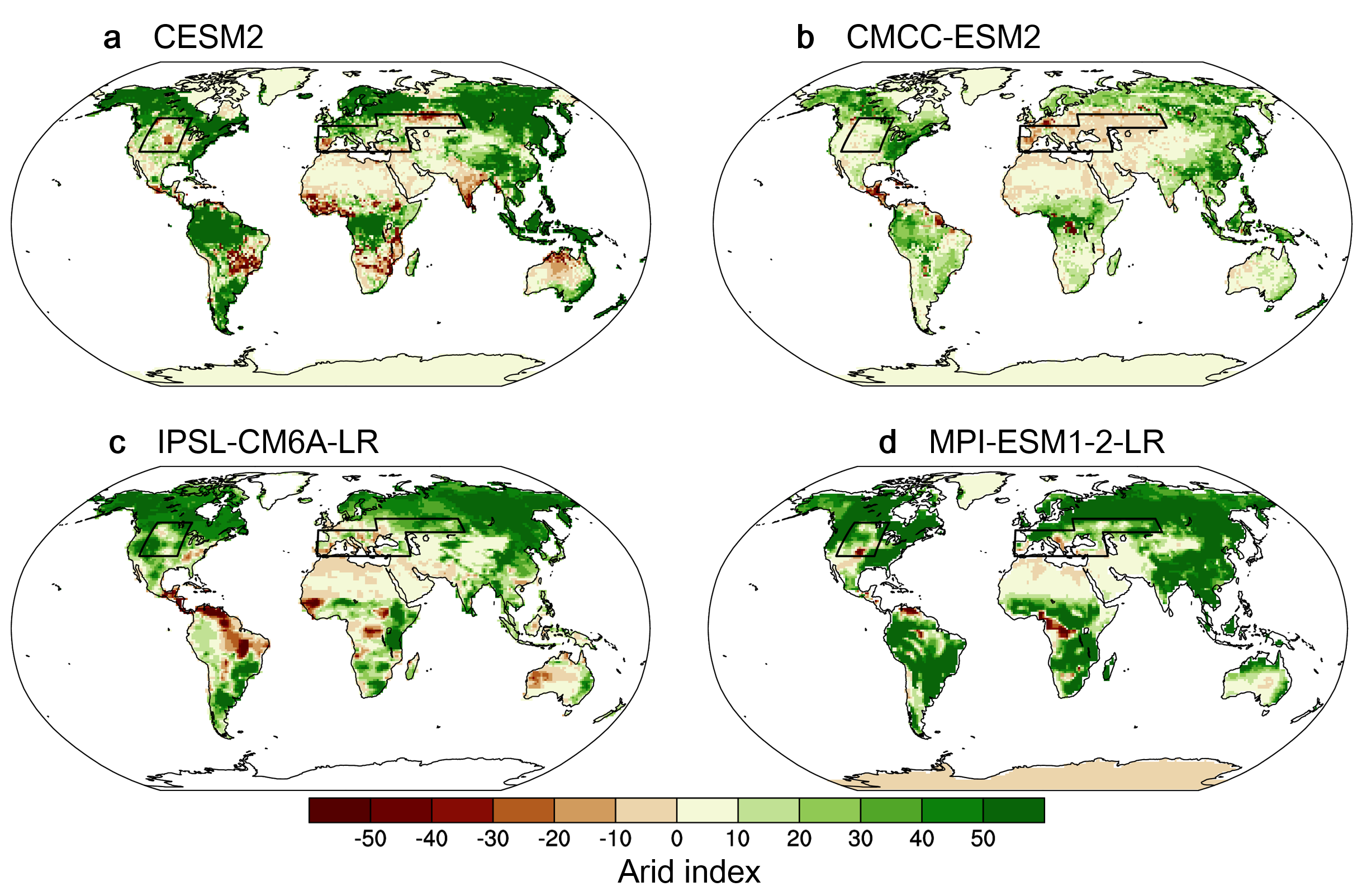


**Fig. S20. Same as Fig. 4b, but for different single model.**


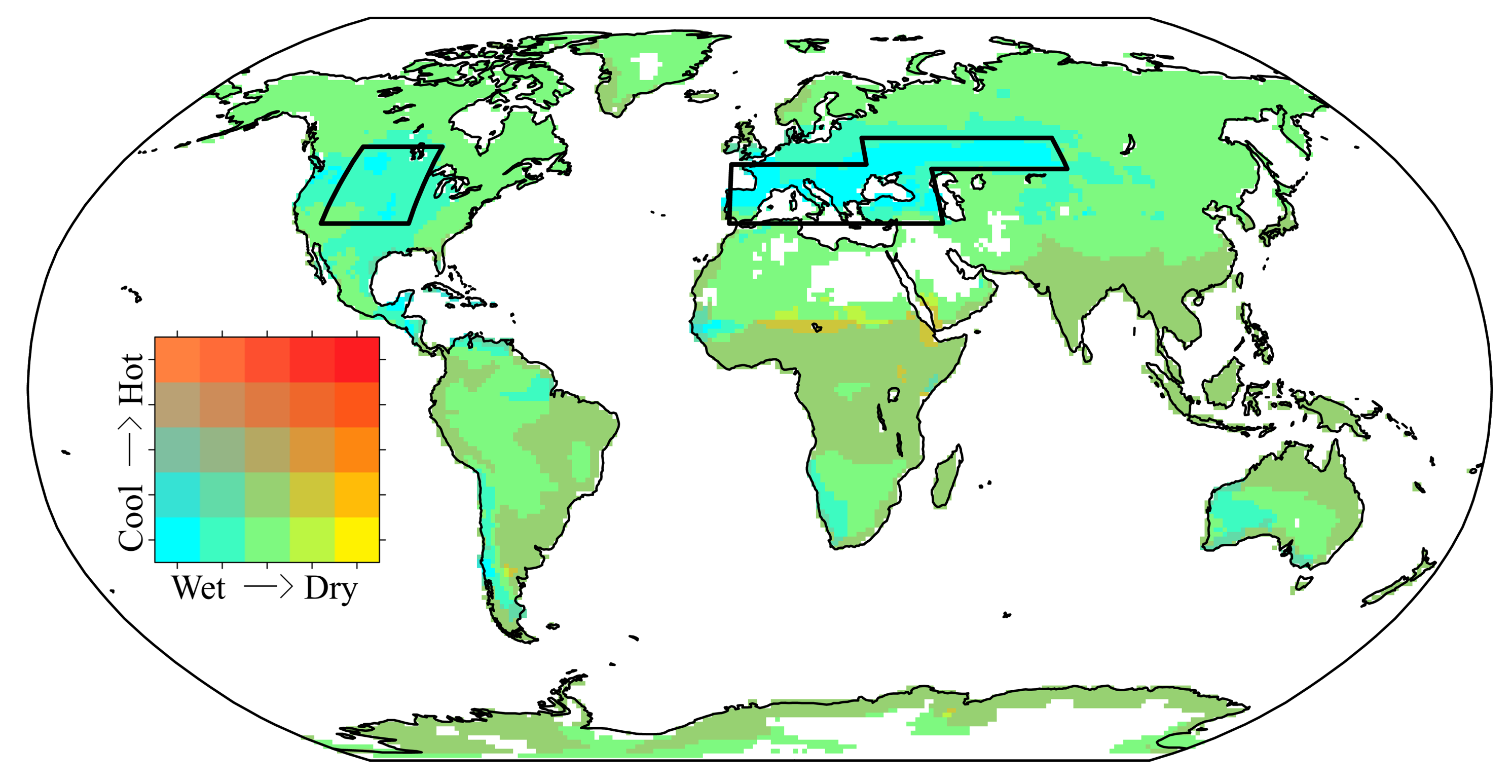


**Fig. S21. Projected influence of emission reduction on surface air temperature (SAT,** **°C) and aridity index for 2060‒2099.** The spatial distribution combines the difference between SSP1-2.6 and SSP5-8.5 in SAT and aridity index, where the differences in SAT corresponding to hot and cool are positive and negative, respectively, and the differences in aridity index corresponding to dry and wet are positive and negative, respectively.


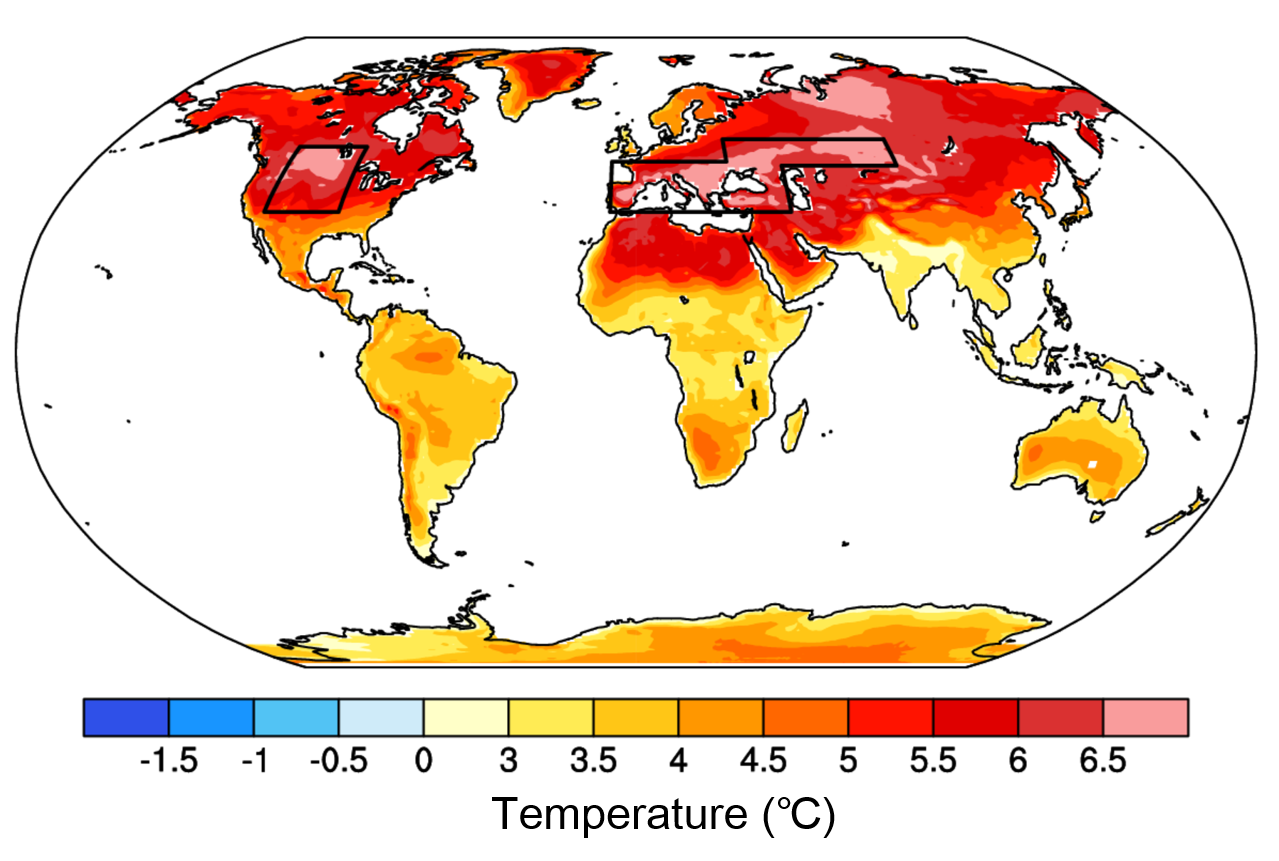


**Fig. S22. Spatial distribution of summer** **surface air temperature (****°C) difference between SSP5-8.5 (2060-2099) and historical (1975-2014) experiments.**


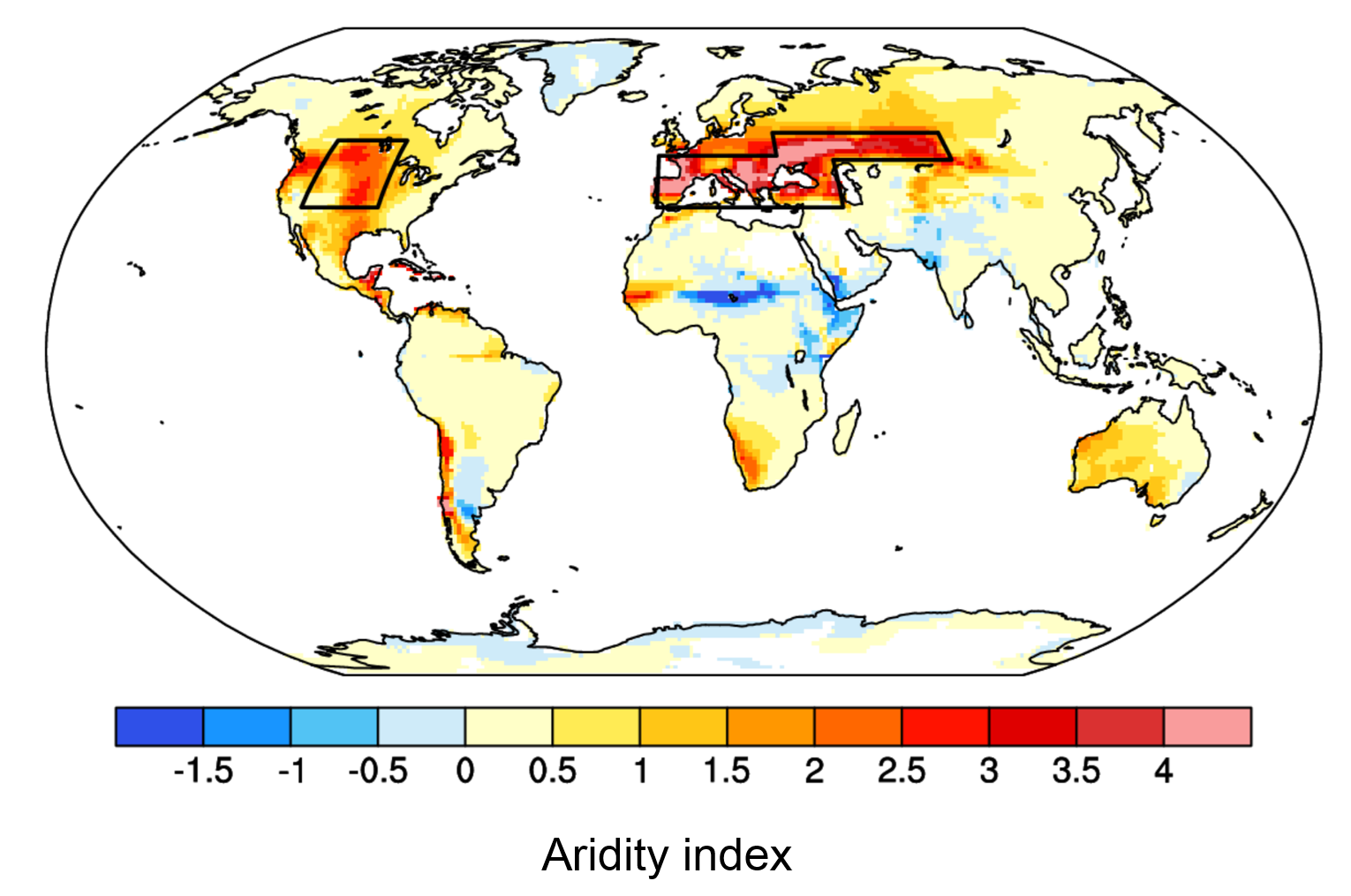


**Fig. S23.Spatial distribution of summer** **aridity index difference between SSP5-8.5 (2060-2099) and historical (1975-2014) experiments.**
